# Climate Change and Agricultural Development: Adapting Polish Agriculture to Reduce Future Nutrient Loads in a Coastal Watershed

**DOI:** 10.1007/s13280-013-0461-z

**Published:** 2013-10-24

**Authors:** Mikołaj Piniewski, Ignacy Kardel, Marek Giełczewski, Paweł Marcinkowski, Tomasz Okruszko

**Affiliations:** Department of Hydraulic Engineering, Warsaw University of Life Sciences, 159 Nowoursynowska Street, 02-776 Warszawa, Poland

**Keywords:** Baltic Sea, SWAT, Agriculture, Climate change, Adaptation measures, Scenario

## Abstract

Currently, there is a major concern about the future of nutrient loads discharged into the Baltic Sea from Polish rivers because they are main contributors to its eutrophication. To date, no watershed-scale studies have properly addressed this issue. This paper fills this gap by using a scenario-modeling framework applied in the Reda watershed, a small (482 km^2^) agricultural coastal area in northern Poland. We used the SWAT model to quantify the effects of future climate, land cover, and management changes under multiple scenarios up to the 2050s. The combined effect of climate and land use change on N-NO_3_ and P-PO_4_ loads is an increase by 20–60 and 24–31 %, respectively, depending on the intensity of future agricultural usage. Using a scenario that assumes a major shift toward a more intensive agriculture following the Danish model would bring significantly higher crop yields but cause a great deterioration of water quality. Using vegetative cover in winter and spring (VC) would be a very efficient way to reduce future P-PO_4_ loads so that they are lower than levels observed at present. However, even the best combination of measures (VC, buffer zones, reduced fertilization, and constructed wetlands) would not help to remediate heavily increased N-NO_3_ loads due to climate change and agricultural intensification.

**Electronic supplementary material:**

The online version of this article (doi:10.1007/s13280-013-0461-z) contains supplementary material, which is available to authorized users.

## Introduction

Aquatic eutrophication caused by excessive loads of biogenic substances transported by rivers to sea waters, is now the primary environmental issue related to the Baltic Sea, a problem that intensified during the 20th century due to rapid economic development in the region (Glasby and Szefer 1998). The increasing scale of eutrophication forced members of society, policy-makers, and researchers to adopt elaborate mechanisms to reduce pollution sources in the Baltic Sea Basin (BSB) and thus prevent further deterioration of sea water quality. Among the proposed measures, the most significant one is the Baltic Sea Action Plan (BSAP) (HELCOM 2007), which combats eutrophication by reducing biogenic substances in rivers discharging into the sea. As one of the BSAP signatories, Poland is obliged to implement a policy that will stimulate activities that will achieve the objectives of this plan. An additional stimulus for implementing measures aimed at reducing nutrient loads in Polish watersheds was the country’s acceptance as a member of the EU in 2004 and the related transposition of regulations resulting from the Water Framework Directive (2000) and the Nitrates Directive (1991), both aimed at achieving the good ecological status of all bodies of water.

The proposed measures seem to be effective, as a reduction of the total nitrogen (TN) and phosphorous (TP) loads reaching the Baltic Sea has been observed at different scales: the entire basin (HELCOM 2011) and the Vistula and Odra basins (Kowalkowski et al. 2012). In Poland, the reduction is predominantly related to a radical modernization of wastewater treatment plants that took place over the past two decades, while Poland’s share of the generated load remains the largest among all the Baltic countries, when expressed in percentages (Kowalkowski et al. 2012). TN and TP loads to the Baltic Sea originate mainly from agricultural diffuse sources (HELCOM 2011), hence European leaders have shifted their focus to agriculture with a number of research programs executed recently (e.g., Baltic Deal, Baltic Compass, Baltic Manure, BERAS Implementation, etc.) in order to achieve environmental targets. However, questions remain concerning the estimation of potential loads related to different alternatives for Polish agricultural development. The future trends related to driving forces, such as climate, land use, demography, global food market demand, competitiveness of Polish farmers in the EU market, and the non-agricultural sector’s demand for land are far from being properly understood and quantified. One of the methods that can be employed to move forward in bringing together and quantifying these trends is multidimensional scenario development, using current state-of-the-art approaches recently set by the SCENES project (Kämäri et al. 2011). Quantification of the potential range of future nutrient loads generated from rural areas as well as the assessment of the efficiency of measures that could be applied in agriculture to combat eutrophication in the forthcoming decades can be done using a combination of scenario studies with mathematical models that are capable of evaluating the effect of changing climate, population, land use, and other important drivers on the state of the environment. This is also a topic about which science could provide needed insight during the policy-making process.

**Fig. 1:**
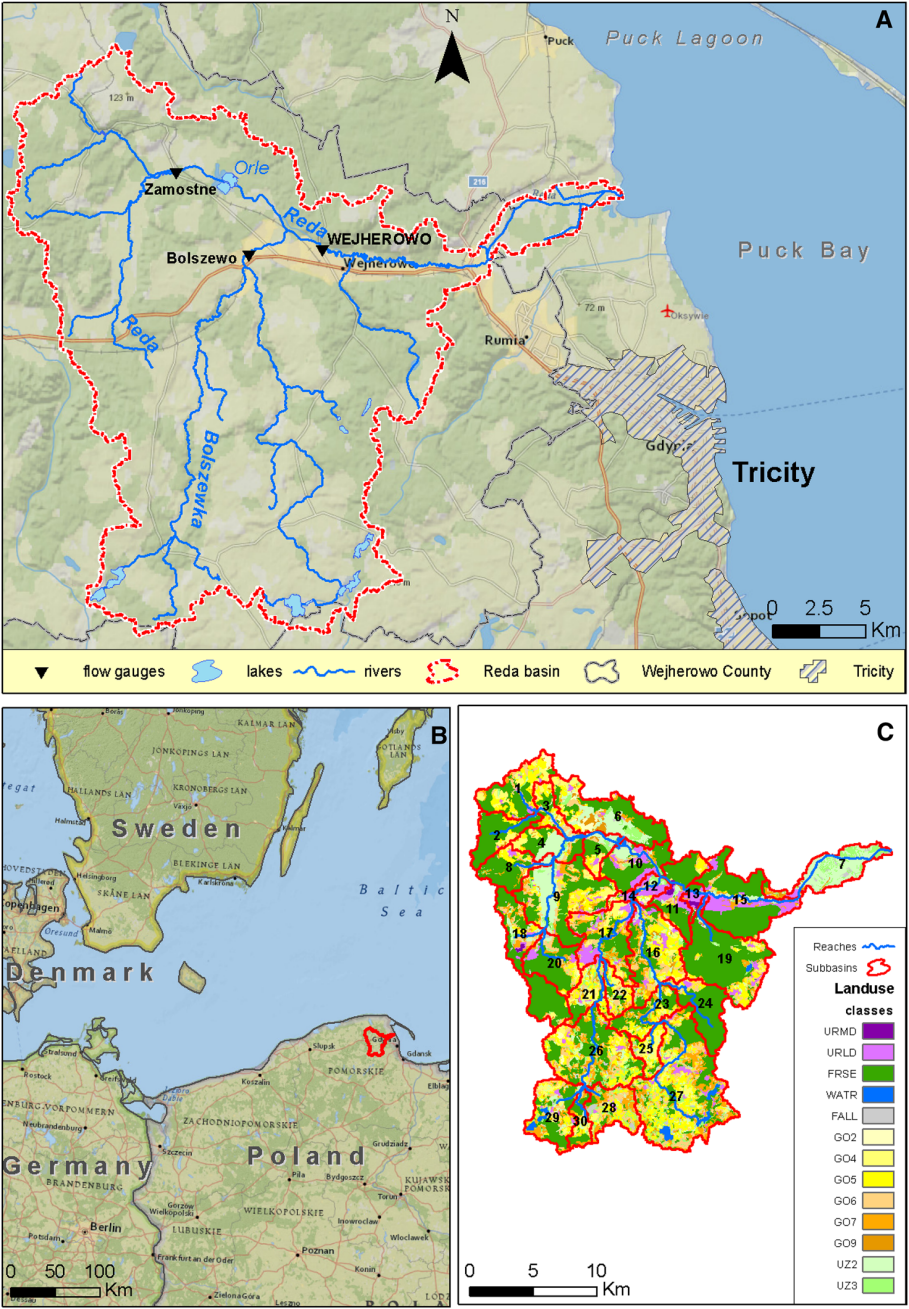
The Reda watershed (**A**), its location in the Baltic region (**B**), and its land cover map (**C**). Key to land use classes: *URMD*—urban residential medium density; *URLD*—urban residential low density; *FRSE*—forests; *WATR*—water; *FALL*—fallow land; GO2–GO9 arable land on soils of different quality; UZ2–UZ3—grassland on soils of different quality

Recent studies using state-of-the-art models carried out at the BSB level (Arheimer et al. 2012; Meier et al. 2012) show quite different results concerning both the effect of climate change on nutrient loads and the efficiency of adaptation measures or best management practices (BMPs). Hence, smaller-scale studies using physically based models that can benefit from more reliable data are required to better understand future impacts in this region. Large-scale studies are of interest to stakeholders and decision-makers at the EU or regional level, while the focus of stakeholders and decision-makers at the provincial level where the decision-making process is often implemented in practice is directed toward small-scale studies. This study was carried out in the Reda watershed situated in northern Poland (Fig. 1), draining an area of 482 km^2^ to the Puck Lagoon (inner Puck Bay, part of the Gulf of Gdańsk). Its objective was to combine state-of-the-art modeling with multidimensional scenario development in order to: (1) quantify nutrient loads under future climate and land use scenarios until 2050 and (2) estimate the effect of agricultural adaptation measures aimed at nutrient load reduction.

## Materials and Methods

### Study Area

The River Reda is situated in northern Poland close to Tricity (Gdańsk, Gdynia, and Sopot), the largest urban zone on the Polish seaside (Fig. 1). Its drainage area is equal to 482 km^2^, which makes it the largest sub-watershed of the Puck Bay watershed. The Puck Lagoon is a very shallow coastal water body, which is particularly sensitive to eutrophication due to its limited water exchange with the outer part of the Puck Bay (Krzymin´ski and Kamin´ska 2005). It belongs to the marine area of the Nadmorski Landscape Park, a designated HELCOM Baltic Sea Protected Area.

Average (1991–2010) daily minimum and maximum temperatures at the nearby IMGW station in Gdynia are −1.5 and 2.9°C for January, and 15.4 and 21.5°C for July, while the annual mean basin-averaged precipitation is 793 mm. The watershed is characterized by a hilly landscape, particularly in its southern part that belongs to the Kashubian Lakeland where the maximum elevation reaches 234 m.a.s.l. and sandy soils dominate the landscape. In the northern part, the Reda-Łeba ice marginal valley is filled with peat deposits that stretch from west to east. Agricultural land occupying 51.2 % of the watershed area is the predominant land cover class, while forests are the second-largest class occupying 41.6 %. The proximity of a large metropolitan area is reflected in the share of urban (mainly low density residential) land which yields 6.6 %.

Most of the farms in studied area are rather small in size [only 28 % of farms are larger than 10 ha, which is still much higher than the average (15 %) in Poland (GUS 2011)]. Mean farm size in Poland is several times smaller than in western European countries in the same climatic zone: Germany, Denmark, and the Netherlands (Banski 2010). Crops cultivated on arable land include the dominant spring cereals on better quality soils in the northwestern part of the basin and extensive farming of winter cereals and potatoes on poor quality soils in the southern part of the basin. The majority of grasslands are cultivated as permanent meadows and pastures. The share of commercial crop production in global agricultural production is estimated to be only 30 %, while the remaining crop production is used mainly for a farm’s livestock feeding. Mean livestock density was estimated at 0.56 LSU ha^−1^ with a dominance of pigs, which is well below 1.5 LSU ha^−1^, the maximum allowed livestock density considered to be not harmful to the environment in the Polish best practices codex.

**Fig. 2:**
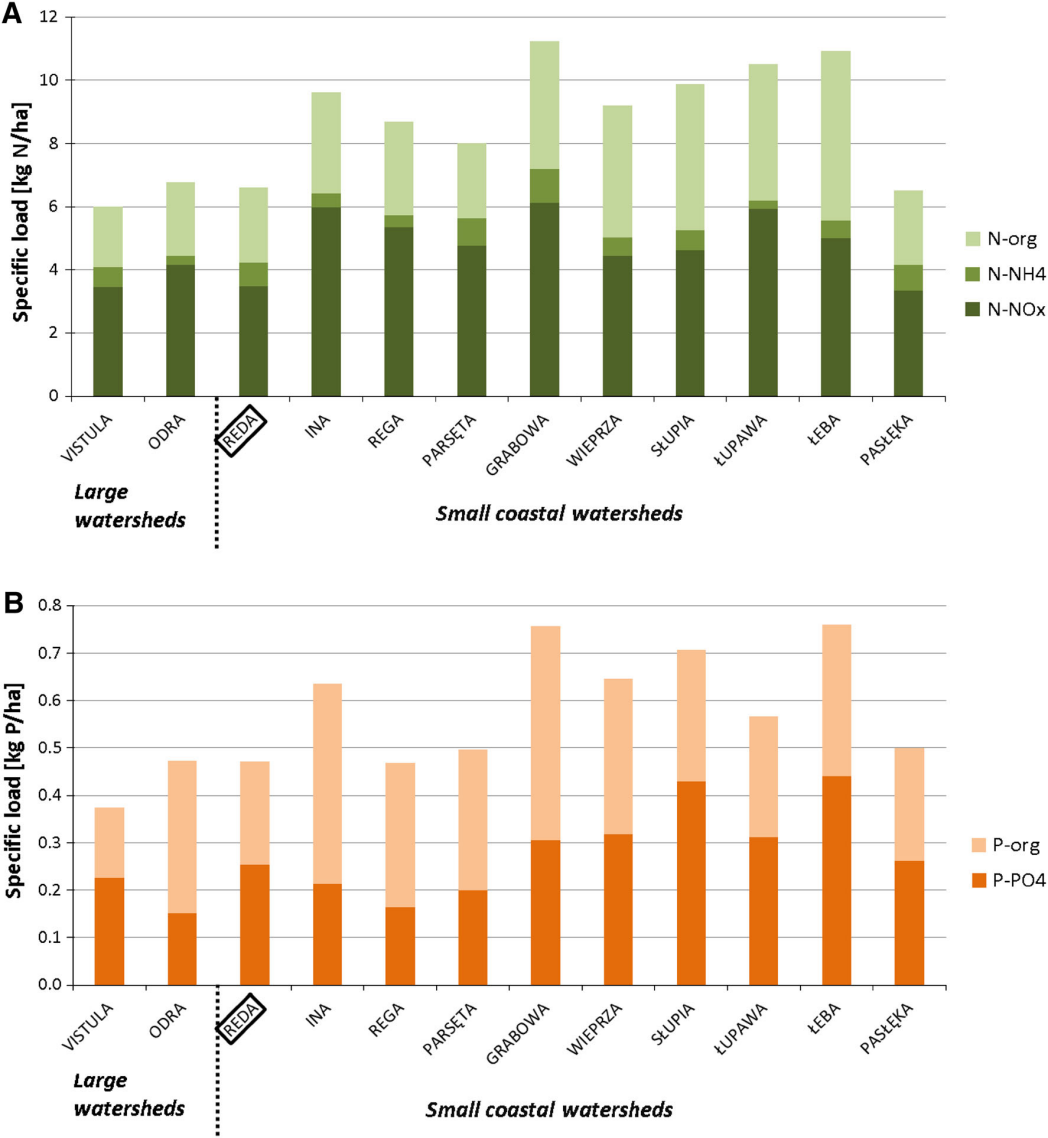
Annual mean specific loads of TN (**A**) and total phosphorous (**B**) from the main Polish rivers to the Baltic Sea in 1993–2001

According to measurements taken by Szymczak and Piekarek-Jankowska (2007), the River Reda contributes ca. 76 % of runoff volume, 72 % of TN, and 65 % of TP to the Puck Bay (Bogdanowicz et al. 2007). TN and TP area-specific loads from the Reda watershed are similar to the loads from the Vistula and the Odra (the largest Polish watersheds representative of the country’s territory), and are significantly lower than the loads from the majority of other coastal watersheds in Poland (Fig. 2). Furthermore, according to the latest Baltic Sea Pollution Load Compilation (HELCOM 2011), area-specific loads from Polish rivers were lower as of 2006 than those shown in Fig. 2 and were relatively small compared to other Baltic states in the case of total N, and relatively large in the case of total P. In particular, they were four times lower than loads from Danish rivers in the case of total N, and 20 % lower in the case of total P, although this is partly caused by lower area-specific runoff of Polish rivers.

The nitrate nitrogen (N-NO_3_) and the phosphate phosphorous (P-PO_4_, or mineral P) are the two most dominant forms of N and P in the Reda waters (Fig. 2; Bogdanowicz et al. 2007). For this reason, we selected these two forms for modeling in this study.

### Modeling Tool

SWAT is a public domain river basin scale model developed to quantify the impact of land management practices in large, complex river basins. For this study, we used the SWAT2009 rev. 528 model (Neitsch et al. 2011). We placed a short description of the model in the Electronic Supplementary Material (ESM), part A.

The SWAT model has proven to be an effective simulation tool, which is manifested by its widespread worldwide use, in particular in the BSB watersheds in Finland (Tattari et al. 2009), Sweden (Ekstrand et al. 2010), Denmark (Hoang et al. 2012), and Poland (Ostojski 2012; Piniewski 2012).

### Model Setup

We performed automatic watershed delineation using a 50 m resolution Digital Elevation Model (DEM) acquired from the Main Centre of Geodetic and Cartographic Documentation (CODGiK) to delineate the Reda watershed into 30 subwatersheds. The mean watershed elevation was equal to 107 m.a.s.l., and 17.4 % of the land was characterized by slopes higher than 10 %. The total length of the streams in the watershed was equal to 143.8 km. A few small lakes with a total capacity below 9 mio. m^3^ situated in the upstream part of the watershed were represented by the pond feature in SWAT, whereas through-flow Lake Orle on the River Reda was modeled as a reservoir.

We used a land cover map from Corine Land Cover (CLC 2006; EEA 2007) in this study. The agricultural land classifications from CLC 2006 were enhanced by the map of soil quality classifications from the Institute of Soil Science and Plant Cultivation in Puławy (IUNG-PIB), defining areas with specific crop requirements and typical fertilization levels. The SWAT input land cover map showing 13 unique land use classifications is presented in Fig. 1C.

We created a soil input map based on soil types and subtypes and a soil layer texture map from IUNG-PIB. We used the total number of 18 unique soil classifications. The dominant soil class is highly permeable sand (usually Cambisols) occupying 51 % of the basin area; 36 % is occupied by different soil types with a texture of loamy sands or sandy loams. Hydrogenic soils, a majority of which are fen peat soils, occupy 12 % of the watershed area.

An intersection of land use map, soil map, and slope classes map in the ArcSWAT interface enabled the creation of 465 HRUs with a mean area of 104 ha. Three slope classes were distinguished: below 2 %, from 2 to 10 %, and above 10 %.

We acquired daily climate data from the Institute of Meteorology and Water Management, Marine Branch in Gdynia (IMGW-PIB) for the time period of 1991–2010. There were five stations with precipitation records, four stations with temperature, humidity, and wind speed records, and one station with solar radiation records. Only one station (Wejherowo) was situated inside the watershed, so all climate data apart from radiation were interpolated at a subbasin level using the Thiessen polygon method prior to using them as input in ArcSWAT (Table 1).

**Table 1:** Calibration and validation goodness-of-fit measures at Wejherowo gaging station (*NSE*—Nash–Sutcliffe Efficiency; *R* ^2^—coefficient of determination; *PBIAS*—percent bias)

Variable	Calibration period			Validation period		
	NSE	*R* ^2^	PBIAS (%)	NSE	*R* ^2^	PBIAS (%)
Discharge	0.75	0.79	−8	0.61	0.78	−18
Sediment load	0.55	0.58	10	0.22	0.23	−12
N-NO_3_ load	0.62	0.62	−4	0.64	0.83	3
P-PO_4_ load	0.53	0.53	−6	−1.78	0.12	7

Sewage waters from Wejherowo, the largest urban area in the watershed, are transferred out of the watershed to the wastewater treatment plant in Gdynia. During a field visit in July 2011, we identified and collected water samples from four active treatment plants in the watershed. Their total daily discharge equal to 950 m^3^ was estimated based on data from the water cadaster available from the Regional Water Management Authority in Gdańsk. Their daily loading of the Total Suspended Sediment (TSS), TN, and TP yields were 125, 33, and 3.7 kg, respectively. Confrontation of these values with discharge and water quality measurements at Wejherowo gaging station shows that point source discharges constitute only 2.3, 4.8, and 4 % of total constituent loads, respectively. We acquired soil chemistry data from 19 agricultural fields within the watershed from the Regional Chemical-Agricultural Station in Gdańsk (OSChR). We used them to estimate initial pools of N and P in soils. The highest concentrations we observed were in organic soils cultivated as grasslands. We acquired wet atmospheric deposition data from the General Inspectorate of Environment Protection (GIOŚ). Mean concentrations of N-NH_4_ and N-NO_3_ yielded 0.66 and 0.36 mg N l^−1^.

Since the focus of this study was the impact of agriculture on water quality, we devoted special attention in the SWAT setup to defining agricultural management practices. We acquired necessary data and expert information from the Pomeranian Agricultural Advisory Board in Gdańsk (PODR) and the Central Statistical Office (GUS 2011, 2012, 2013). We collected all the necessary data at the commune level, the smallest administrative unit in Poland. The area delineated by the four largest communes overlapping with the watershed covers 82 % of agricultural land within the watershed, which is assumed to be satisfactory from the point of view of data representativeness.

Defining crop structure in SWAT requires some generalization of official statistics. We defined seven major crops grown on arable land: winter cereals (rye), spring cereals (oats and spring wheat), potatoes, field peas, red clover, and spring canola. Cereals and potatoes, cultivated in a traditional extensive manner rather than in an intensive manner, constituted nearly 90 % of total arable land area. Mean mineral fertilizer usage in 1998–2006 yielded 40 kg N ha^−1^ and 11 kg P ha^−1^; however, real doses used by farmers are crop-dependent and presumably higher, as given figures refer to the total area of agricultural land, which includes, e.g., fallow land and grassland, where mineral fertilizers are never or rarely used. Organic fertilizers used by farmers in the Reda watershed include solid manure and slurry. They are predominantly spread on grassland fields and potato fields (only solid manure). We verified organic fertilizer doses against livestock data from official statistics.

### Model Calibration

We placed a full description of the calibration strategy in the ESM, part B. A short summary is given below. We calibrated and validated SWAT using daily discharge data at three gages: Bolszewo, Zamostne, and Wejherowo, and using bimonthly TSS, N-NO_3_, and P-PO_4,_ loads measured at Wejherowo station. We acquired discharge data from IMGW-PIB and water quality measurement data from GIOŚ. The calibration and validation periods were 1998–2002 and 2003–2006, respectively. We applied the SUFI-2 automatic calibration tool from SWAT-CUP software (Abbaspour 2008). We set the Nash–Sutcliffe Efficiency (NSE) as an objective function (Moriasi et al. 2007). Additionally, we evaluated best simulations using *R* ^2^ and percent bias (PBIAS), and characterized the whole set of good simulations in terms of the uncertainty measures (cf. ESM; Abbaspour 2008).

### Future Scenario Assumptions

For this study, we selected the period centered around 2050 as the time horizon of future scenarios. Two major driving forces of future watershed change are climate and land use. In this context, climate change is understood as a projected change in precipitation and temperature (from a given climate model) as well as in atmospheric CO_2_ concentration (from a given emission scenario driving a climate model). Land use change is understood here as any alteration of human utilization of land surface and thus is not limited to land cover change, but can also include, for example, intensification of agricultural production. While climate change projections for the Reda watershed were downscaled from a climate model, general assumptions of land use change were established in a framework of the Baltic Compass project. Within this project two National Round Tables gathering agricultural researchers, policy-makers, and practitioners were organized in 2011 and 2012 in Poland. Additionally, three workshops focused on the Reda watershed, involving both experts from local extension services (in Gdańsk office and Wejherowo branch) as well as local water management authorities (IMGW-PIB in Gdynia) were organized in 2011 and 2012. Modeling results were being communicated to experts and stakeholders at each stage of the process; hence, the approach of creating these scenarios followed to some extent methodology previously outlined in SCENES (Giełczewski et al. 2011; Kämäri et al. 2011).

**Fig. 3:**
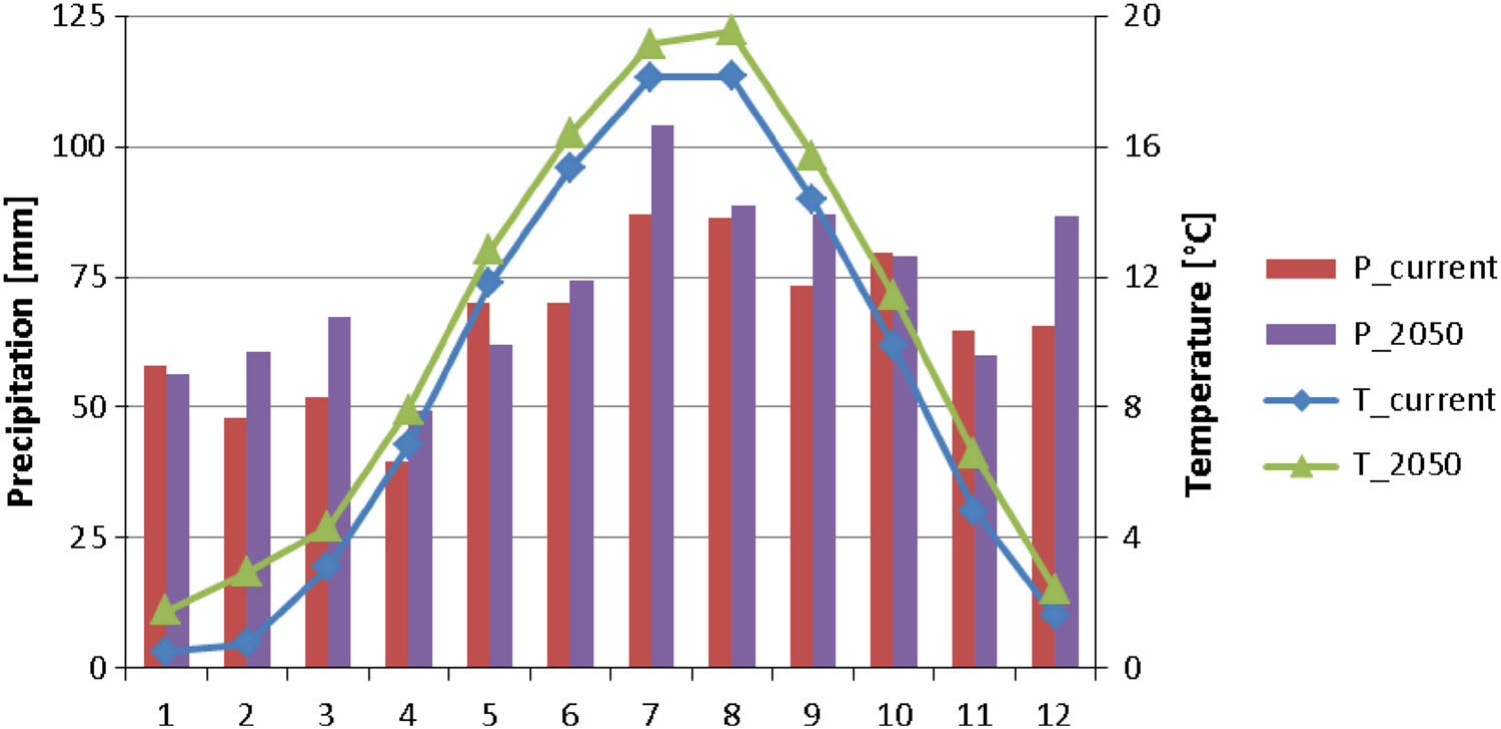
Basin-averaged downscaled projections of monthly precipitation (P) and temperature (T) for 2050s versus current situation in the Reda watershed

We acquired the climate projections by ECHAM5 GCM driven by the SRES A1B emission scenario and coupled with RCA3 RCM from the Swedish Hydrological and Meteorological Institute (SHMI) (Samuelsson et al. 2011). The A1B scenario assumes a future world of very rapid economic growth, low population growth, and rapid introduction of new and more efficient technology. This scenario has been selected, as it assumes a balanced emphasis on all energy sources and its projections of future greenhouse gas emissions and concentrations by 2050 are in the middle of the range of SRES family projections. We used output data from two 50-by-50 km grid points from the RCA3 model overlapping with the Reda watershed. We applied the delta change approach to represent the future climate in SWAT (Fowler et al. 2007). First, we calculated the delta factors using the RCM output data for two time periods, one representing the current climate (1984–2013) and one representing the future climate (2035–2064). We calculated precipitation monthly factors multiplicatively and temperature additively. Second, we used calculated delta factors as input in SWAT. Figure 3 illustrates basin-averaged downscaled projections of monthly precipitation and temperatures for the 2050s versus those in the current situation. The mean annual temperature increase equals 1.3°C and varies from 0.8°C in December to 2.2°C in February. The projected annual precipitation increase yields 80 mm (10 %), although in several months there is a projected decrease in precipitation.

Apart from changes in climate variables, according to RCM projections, we made two additional changes to better represent the future climatic conditions. We estimated future atmospheric CO_2_ concentration (ppm) based on projections of greenhouse emissions associated with the SRES A1B scenario. A 50 % increase in CO_2_ was used as an input SWAT parameter representing future conditions. Furthermore, we adjusted two SWAT parameters to better account for changes in plant physiological parameters driven by an increase in CO_2_ concentration than in the original SWAT code: the stomatal conductance GSI and the maximum leaf area index BLAI (cf. Piniewski 2012 for more details).

While climate change in the Reda watershed is driven mainly by global-scale factors, land use change is related to factors acting on various scales: climate, population growth, future national and EU policies (in such domains as agriculture and spatial planning), local and global food demand, etc. Projections of land cover change due to population growth (or decline) are probably the least uncertain ones. The study area has experienced a rapid growth of urban sprawl in recent years: According to data from the Central Statistical Office (GUS 2012) mean annual population growth from 1995 to 2011 was 3 % in rural areas and 0.3 % in urban areas. Furthermore, the highest annual growth rate, up to 4 %, was found in communes situated closest to the Tricity metropolitan area. According to stakeholders, this growth caused two types of land cover change: (1) urban sprawl of the cities (Wejherowo and Reda) and (2) transformation of marginal land in rural areas into residential areas. This trend is supposed to continue in the foreseeable future: In Wejherowo County, covering ca. 90 % of the Reda watershed, the expected mean annual population growth from 2011 to 2035 is 0.85 % in urban areas and 1.27 % in rural areas (GUS 2012). These figures were extrapolated until 2050 and transformed into urban land cover growth, as land cover change in contrast to population growth can be directly represented in SWAT. The calculated value of the future increment in the area of low density residential land cover (cf. Fig. 1C) yielded 909 ha, which is 30 % of the current area of this land cover class (cf. ESM, part C for details on calculation of this value).

**Fig. 4:**
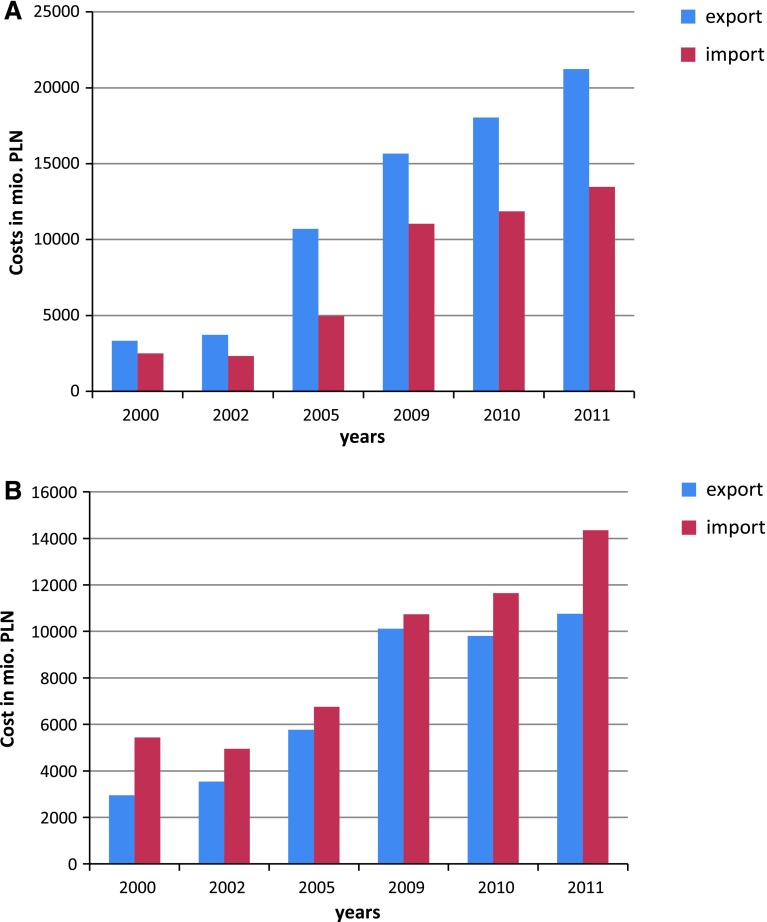
Foreign trade of agricultural products in Poland (GUS 2013): **A** Livestock and animal products. **B** Main plant-based farm and food products

While urban sprawl in the Reda watershed seems inevitable, there is uncertainty related to the future of agriculture. Therefore, on the top of urban land cover change, we developed two agricultural scenarios for this area: one assuming spontaneous development of agriculture and the second its rapid intensification. The first scenario—hereafter referred to as the Business-as-Usual (BAU-2050)—assumes adaptation of production to rising temperatures (e.g., earlier sowing dates, longer growing periods) and takes into account some of the recently observed trends (e.g., biogas plants using corn silage as a substrate); however, crop structure, animal production, and fertilizer usage remain either unchanged or only slightly altered compared to the reference state. In contrast, the second scenario—hereafter referred to as the Major Shift in Agriculture (MSA-2050)—assumes that Poland (and the Reda watershed in particular) will experience a major change in agriculture. A rapid growth in the export of Polish agricultural products in recent years (Fig. 4) accompanied by a considerable growth in the share of commercial production in global agricultural production increased from 62 % in 1990 to 70 % in 2006 (from 62 % in 1990 to 70 % in 2006; Bański 2010) form a background for this scenario. Furthermore, the growth potential of Polish agriculture has not yet been depleted, because in 2008, its labor productivity was 3.5 times lower than in the European Union (EU-27; Poczta et al. 2012). New conditions for rural development now exist under the Common Agricultural Policy, so in the future, Polish agriculture might resemble the intensive agriculture of some of its neighboring Western countries like Denmark, Germany, or Sweden. In order to create a coherent and plausible scenario and for practical reasons, we selected one country, Denmark, as a good model for what Poland will ultimately resemble. We think that both scenarios form a range of possible changes that Polish agriculture is likely to undergo in the future: a shift into Danish-type intensive agriculture in the Reda watershed is the upper limit of possible changes, whereas the BAU scenario is its lower limit. We made the following assumptions in developing the MSA-2050 scenario:

- We acquired agricultural statistical data for the year 2010 for Denmark (DST 2011) and the main communes of the Reda watershed (GUS 2011);
- We adapted the livestock structure and density from Danish conditions in order to assess the availability of organic fertilizers;
- We adapted the crop structure from Danish conditions, assuming that the total agricultural area would be the same as in BAU-2050; increasing the import of fodder (Fig. 4) supports this assumption;
- Due to the substantial availability of organic fertilizers, we reduced the utilization of mineral fertilizers and replaced them with organic ones (mainly slurry).
- We adapted fertilizer rates from Danish standards (MF 2011) and fit them to the soil structure and application timing present in the SWAT setup of the Reda watershed. Even though these rates are several times higher than those applied at present, there are areas in Poland where such rates of slurry are applied (Soroko and Strzelczyk 2009).

The quantitative characterization of scenario assumptions and their representation in the SWAT setup is presented in Table 2. Developed agricultural scenarios were represented in SWAT by modifications of HRU tables with scheduled management practices.

**Table 2:** Comparison of two developed agricultural scenarios for the Reda watershed in 2050s

Scenario feature	BAU-2050	MSA-2050
Population density	Increase from 153 people per km^2^ to 202 people per km^2^ (by 32 %)	
Urban population	Decrease from 58 % in 2011 to 56 % in 2050	
Type of agriculture	Traditional (extensive), oriented on own farm needs (driven by local socio-economic conditions and EU policies)	Intensive, export-oriented (driven by global food demand)
Crop structure (% of agricultural land; SWAT crop names used)	Tall fescue (grassland) 25.9 %, oats 22.2 %, rye 16.9 %, spring wheat 13.9 %, potatoes 9.2 %, red clover 6.1 %, corn silage 2.4 %, field peas 2.2 %, spring canola 1.2 %	Spring wheat 26.0 %, spring barley 22.8 %, tall fescue (grassland) 15.6 %, red clover 12.1 %, Corn silage 9.5 %, spring canola 6.3 %, potatoes 3.0 %, field peas 2.2 %, rye 2.0 %, oats 0.5 %
Livestock density	0.56 LSU ha^−1^	1.43 LSU ha^−1^
Fodder source	Locally produced fodder	Imported fodder
Fertilizer types and rates on agricultural land	Mineral fertilizer (N 66 %, P 80 %), organic fertilizer (N 34 %, P 20 %) Average rate 37 kg N ha^−1^, 12 kg P ha^−1^	Mineral fertilizer (N 23 %, P 8 %), organic fertilizer (N 77 %, P 92 %) Average rate 102 kg N ha^−1^, 53 kg P ha^−1^
Timing of practices	Shift triggered by warmer climate	Shift triggered by warmer climate
Expected yields	Similar to current ones	Much higher than current ones
Expected water pollution	Similar to current state	Much higher than current state

### Adaptation Measures

The starting point for selecting proper adaptation measures was the list of prioritized measures elaborated by the Baltic COMPASS project.^1^ These reports constitute the most up-to-date knowledge on utilization of BMPs in the Baltic countries. The list contains 25 different measures mainly focused on reducing nutrient losses from agriculture at different stages of production. The final selection of measures, made with stakeholders’ advice, was a trade-off forced by model limitations (not all interesting measures can be represented in SWAT). Finally, four measures were selected as valuable for stakeholders and possible for modeling in SWAT (cf. Tattari et al. 2009; Lam et al. 2011; Laurent and Ruelland 2011; Glavan et al. 2012):

1. Vegetative cover in autumn and winter (VC)
2. Buffer zones along water areas and erosion-sensitive field areas (BZ)
3. Avoiding fertilization in risk areas (RA)
4. Constructed wetlands for nutrient reduction/retention (CW)

In order to implement measure VC, that is supposed to have an anti-erosive function as well as to reduce nitrogen leaching to groundwater, we made modifications in scheduled management practices in the model structure. Red clover was used as a catch crop after harvest of spring cereals and corn silage, whereas rye was used as a catch crop after harvest of potatoes. This measure was defined in 47.4 and 55.8 % of agricultural land in BAU-2050 and MSA-2050, respectively.

We implemented measure BZ using the vegetative filter strip (VFS) sub-model of SWAT (Neitsch et al. 2011) that uses different empirical reduction rate equations (White and Arnold 2009). VFSs were defined in all agricultural land HRUs. The drainage area to VFS area ratio in an HRU was defined as 10. VFS function was activated in 100 % of agricultural land.

We represented measure RA in the model as follows: Fertilizer rates were reduced by 50 % in the schedule of management practices in SWAT in selected HRUs referred to as “Risk area” HRUs (those HRUs with agricultural land use that satisfied at least one of the following features: (1) slopes above 10 %; (2) tile drainage operation; (3) heavy soils. This measure was defined in 19.6 % of agricultural land).

We represented measure CW in SWAT using its wetland function, in which sediment and nutrients are removed in wetlands by settling (Neitsch et al. 2011). This function was activated in all sub-basins with a share of agricultural land use above 50 %. A wetland area was defined to be 1 % of the agricultural area in the respective sub-basin, while the volume was calculated assuming a 1 m storage depth. The fraction of sub-basin area draining to the wetland area was defined as 10 %, since it is assumed that CWs are small water bodies built in upstream areas in small ditches and creeks or in natural depressions. This measure was defined in 0.96 % of agricultural land (in terms of land occupied by a wetland area). However, in reality, it affected 10 % of the agricultural area—the whole area draining to the wetland.

Measure CW was the only one that was tested only under the MSA-2050 scenario since it was assumed that under BAU-2050 CW will not gain much more popularity in Poland than they have today. At present, CWs are not used by Polish farmers to retain nutrients flushed from fields, while they are sometimes used as small-scale wastewater treatment plants.

### Model Scenario Design

Two possibilities of future climate (Current or ECHAM5-RCA3-SRESA1B climate) combined with three possibilities of future land use (Current, BAU, or MSA land use) produced six unique combinations of model experiments (Table 3). Such an experimental design allows one to study single effects (climate or land use) and combined effects (climate and land use). In the next step, adaptation measures were implemented into two combined future scenarios: BAU-CC-2050 and MSA-CC-2050 (Table 3).

**Table 3:** Model scenario design

Driving forces	Climate		
	Current	2050 (ECHAM5-RCA3-SRESA1B)	2050 (ECHAM5-RCA3-SRESA1B) + adaptation measures
Land use			
Current	1. Baseline	4. CC-2050	
2050 (Business-As-Usual)	2. BAU-2050	5. BAU-CC-2050	7. BAU-CC-2050 + VC 8. BAU-CC-2050 + BZ 9. BAU-CC-2050 + RA 10. BAU-CC-2050 + All
2050 (MSA)	3. MSA-2050	6. MSA-CC-2050	11. MSA-CC-2050 + VC 12. MSA-CC-2050 + BZ 13. MSA-CC-2050 + RA 14. MSA-CC-2050 + CW 15. MSA-CC-2050 + All

For each model experiment, we calculated four variables (model outputs):

- Mean annual discharge to the Puck Lagoon (*Q*);
- Basin-averaged mean annual N-NO_3_ leaching past the bottom of soil profile to shallow groundwater aquifer (*NG*);
- Mean annual N-NO_3_ load to the Puck Lagoon (*N*);
- Mean annual P-PO_4_ load to the Puck Lagoon (*P*).

Comparison of model runs 2–6 with model run 1 enables the study of single or combined effects of climate and land use change on the abovementioned variables. For a variable *X* (that can be substituted by *Q*, *NG*, *N*, or *P*), a respective indicator Δ*X*_1_ was calculated as follows:

1 $$ \Updelta X_{1} = \frac{{X_{\text{scenario}} - X_{\text{baseline}} }}{{X_{\text{baseline}} }} \times 100\,\% . $$

Comparison of model runs 7–10 with model run 5 or model runs 11–15 with model run 6 enables one to study the efficiency of adaptation measures under future climate and land use conditions. For a variable *X*, a respective indicator Δ*X*_2_ was calculated as follows:

2 $$ \Updelta X_{2} = \frac{{X_{\text{adaptation}} - X_{\text{scenario}} }}{{X_{\text{scenario}} }} \times 100\,\% $$

Finally, comparison of model runs 7–15 with model run 1 enables one to assess what the combined effect of climate change, land use change, and applying an adaptation measure will be compared to the reference state. For a variable *X*, we calculated a respective indicator Δ*X*_3_ as follows:

3 $$ \Updelta X_{3} = \frac{{X_{\text{adaptation}} - X_{\text{baseline}} }}{{X_{\text{baseline}} }} \times 100\% . $$

## Results

### Reference Conditions

The full description of the model calibration and validation results is given in the ESM, part D, while a short summary is given below. The values of goodness-of-fit measures at Wejherowo indicated good model performance for discharge and N-NO_3_ in both calibration and validation periods (Table 1). However, for TSS and P-PO_4_ load, the results for the validation period were remarkably worse than the results for the calibration period. We observed that the measured N-NO_3_ concentrations had a very similar seasonal pattern in calibration and validation periods in contrast to TSS and P-PO_4_ concentrations. We discuss this issue in further detail in the ESM. Despite low *R* ^2^ and NSE in the validation period, acceptable values of PBIAS and of the uncertainty measures in the validation period indicated that the model can be used for scenario simulations of P-PO_4_ loads.

We wrote parameters related to the best simulation found using SUFI-2 (cf. ESM, Tables S1–S4) into the model and executed a 20-year-long simulation run (1991–2010, including a 3-year warm-up period). This simulation run defined the baseline scenario, representing the current conditions in the Reda watershed (Table 4). Because there was a very low proportion of surface runoff in water yield (8 %) we found that an area-specific load of N-NO_3_ in surface runoff was only 0.44 kg N ha^−1^ year^−1^, compared to 2.29 and 1.15 kg N ha^−1^ year^−1^ in subsurface runoff (this includes outflow from drainage ditches) and baseflow, respectively. This difference can be explained by high percolation of dominating sandy soils contributing to high N-NO_3_ leaching into the shallow aquifer, and by relatively high slopes in the study area contributing to high N-NO_3_ loading in subsurface runoff. Mean annual TSS loads were in accordance with other data sources (Szymczak and Piekarek-Jankowska 2007) as were mean nutrient loads^2^ at the watershed outlet.

**Table 4:** Average annual basin values of water and nutrient balance in the baseline period

Variable	Value
Water balance (mm)	
Precipitation	793
Snowmelt	58
Surface runoff	25
Subsurface runoff	44
Tile drainage	14
Baseflow	206
Total runoff	299
Percolation to the shallow aquifer	250
Actual evapotranspiration	455
PET	684
Area-specific loads (kg ha^−1^ year^−1^)	
Sediment (denudation index)	524
Organic N	1.41
Organic P	0.28
N-NO_3_ in surface runoff	0.44
N-NO_3_ in subsurface runoff	2.29
Soluble P	0.03
N-NO_3_ leaching to groundwater	29.3
N-NO_3_ in baseflow	1.15
Annual loads at watershed outlet (t year^−1^)	
TSS	2.24
N-NO_3_	168
P-PO_4_	16.3

Organic N and P were not calibrated, so presented values are indicative only (they are underestimated compared to measured values)

### Nutrient Loads in Future Scenarios

**Fig. 5:**
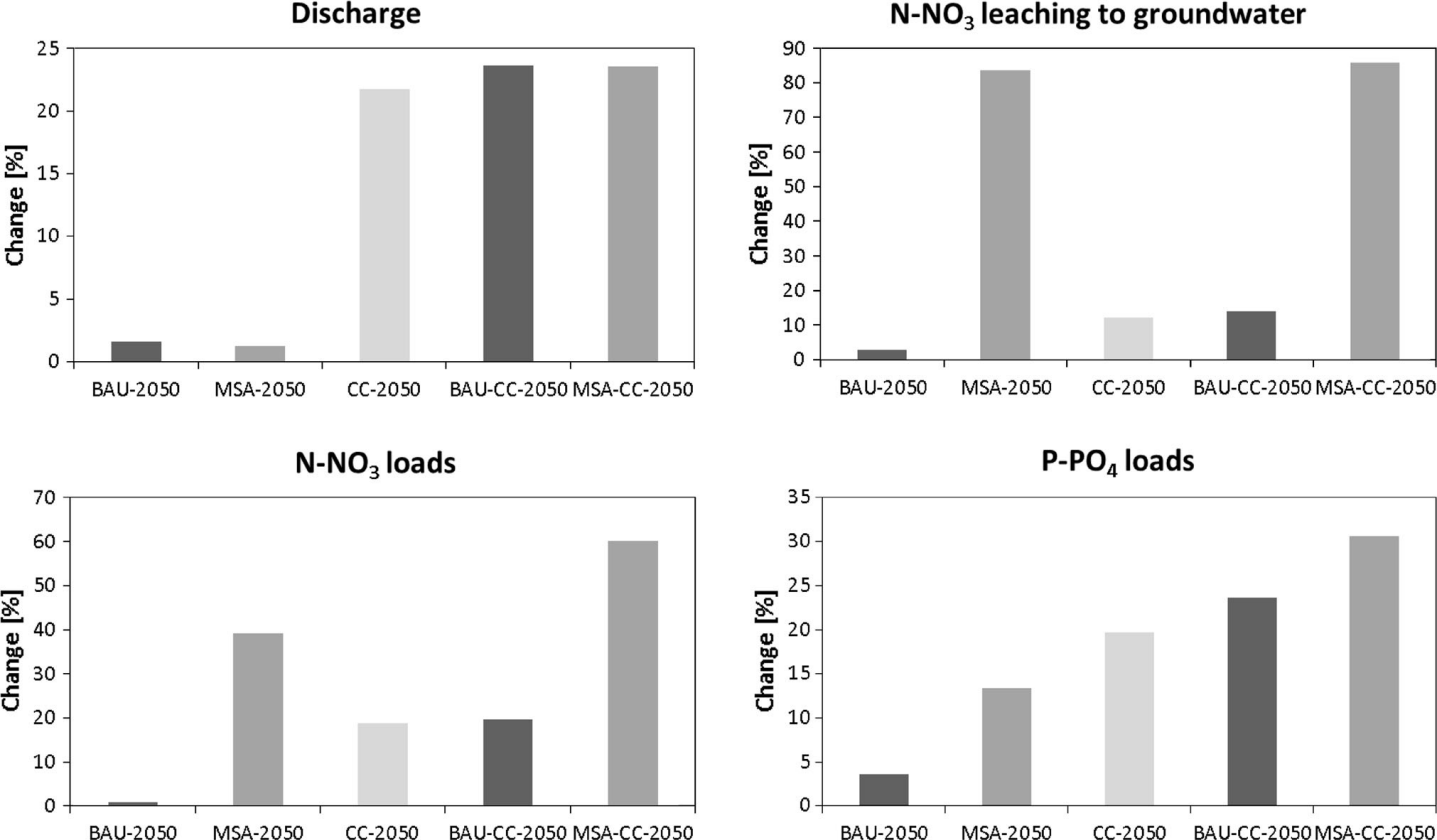
Percent changes in selected indicators for analyzed scenarios in 2050 with respect to current (baseline) scenario

The results presented in Fig. 5 (calculated using Eq. 1) show that under the BAU scenario without considering climate change (BAU-2050), nutrient loads and leaching remain on a similar level as that occurring at the present time. In contrast, large changes are expected under the Major Shift in Agriculture scenario (MSA-2050). While both BAU-2050 and MSA-2050 are rather neutral regarding water balance, under future climate (CC-2050,) mean discharge is expected to rise by 22 %, which has a direct effect on projected loads. This increase can be attributed mainly to projected precipitation increase, as projected temperature increase is rather moderate under the A1B scenario (cf. Fig. 3) and causes mainly the advancing date and reduced amount of spring snowmelt. Rising CO_2_ causes a decrease in potential evapotranspiration (PET), whose magnitude is larger than an increase in PET caused only by rising temperature.

However, the effect of intensified agriculture is clearly larger than the effect of climate change. The results for all studied indicators are amplified when combined effects of climate and land use change are analyzed: For example, under MSA-CC-2050, N-NO_3_ and P-PO_4_ loads are expected to rise by 60 and 31 %, respectively.

**Fig. 6:**
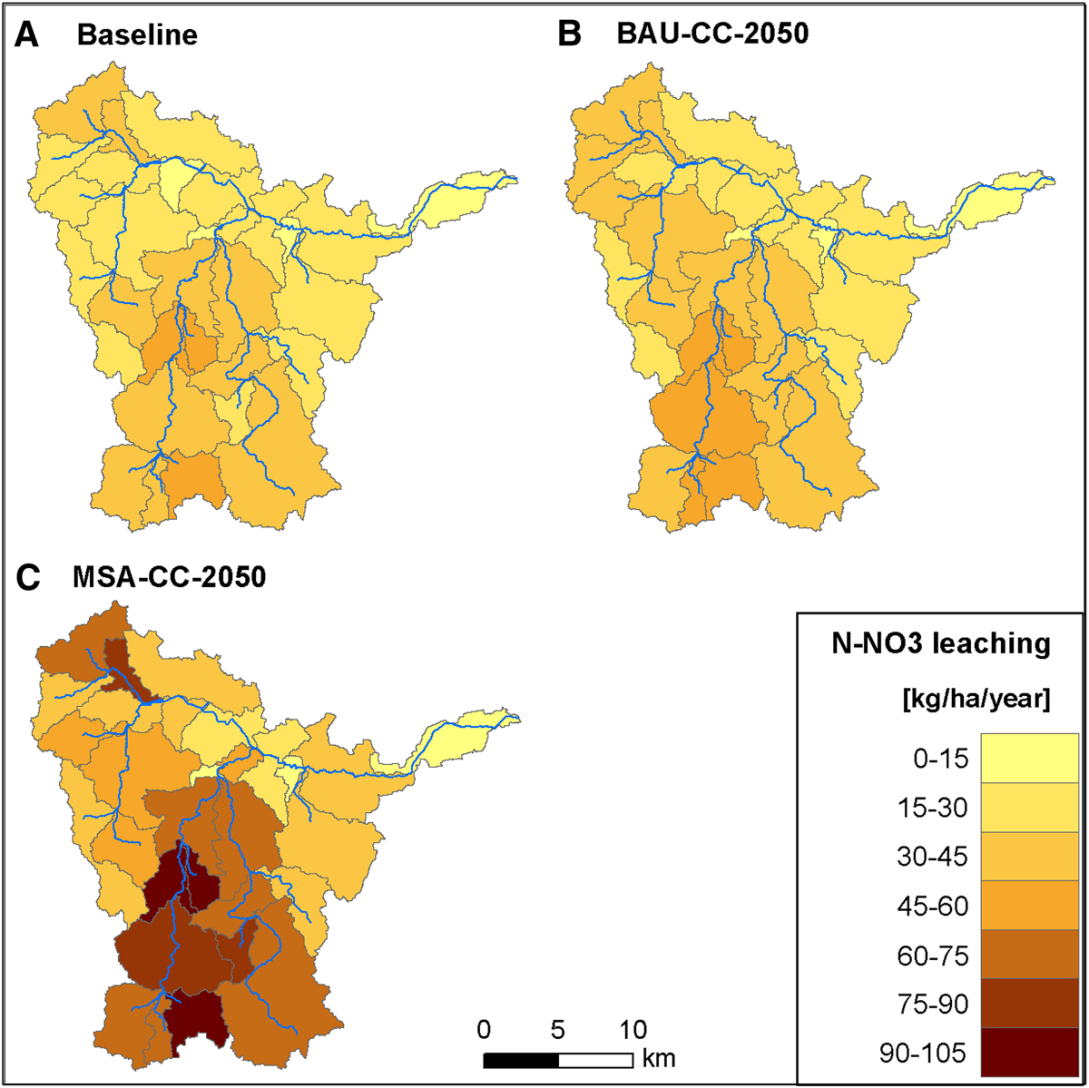
Spatial variability at subbasin level in mean annual N-NO_3_ leaching to the shallow groundwater aquifer in the baseline period (**A**) and under two future scenarios: BAU under climate change (**B**) and MSA under climate change (**C**)

A direct comparison of BAU-CC-2050 with MSA-CC-2050 shows that under the latter, nitrate water pollution is expected to grow significantly as a result of agricultural intensification (Fig. 5). Figure 6 illustrates the spatial variability of N-NO_3_ leaching to the shallow groundwater aquifer for these scenarios. Comparing these maps with the land cover map (Fig. 1C) demonstrates that in sub-basins with a high share of arable land, N-NO_3_ leaching has increased more than twofold. Such a high increase is well in agreement with the field experiments conducted on loamy sands of southwestern Poland (Soroko and Strzelczyk 2009). They showed that the rate of slurry application has a very strong effect on N-NO_3_ concentration in groundwater and drainage water which can be explained by the fact that light soils are more susceptible to high N-NO_3_ leaching due to low denitrification. In the case of P-PO_4_, the difference between BAU-CC-2050 and MSA-CC-2050 is much smaller than for N-NO_3_. It is worth noting that simulated yields and harvest of main crops are expected to be higher by 31 and 49 % in MSA-CC-2050 compared to BAU-CC-2050, respectively, which illustrates another facet of the problem we studied. The highest increase in yield in Danish-model agriculture compared to traditional, extensive agriculture is expected for corn silage (51 %).

### Efficiency of Adaptation Measures

**Fig. 7:**
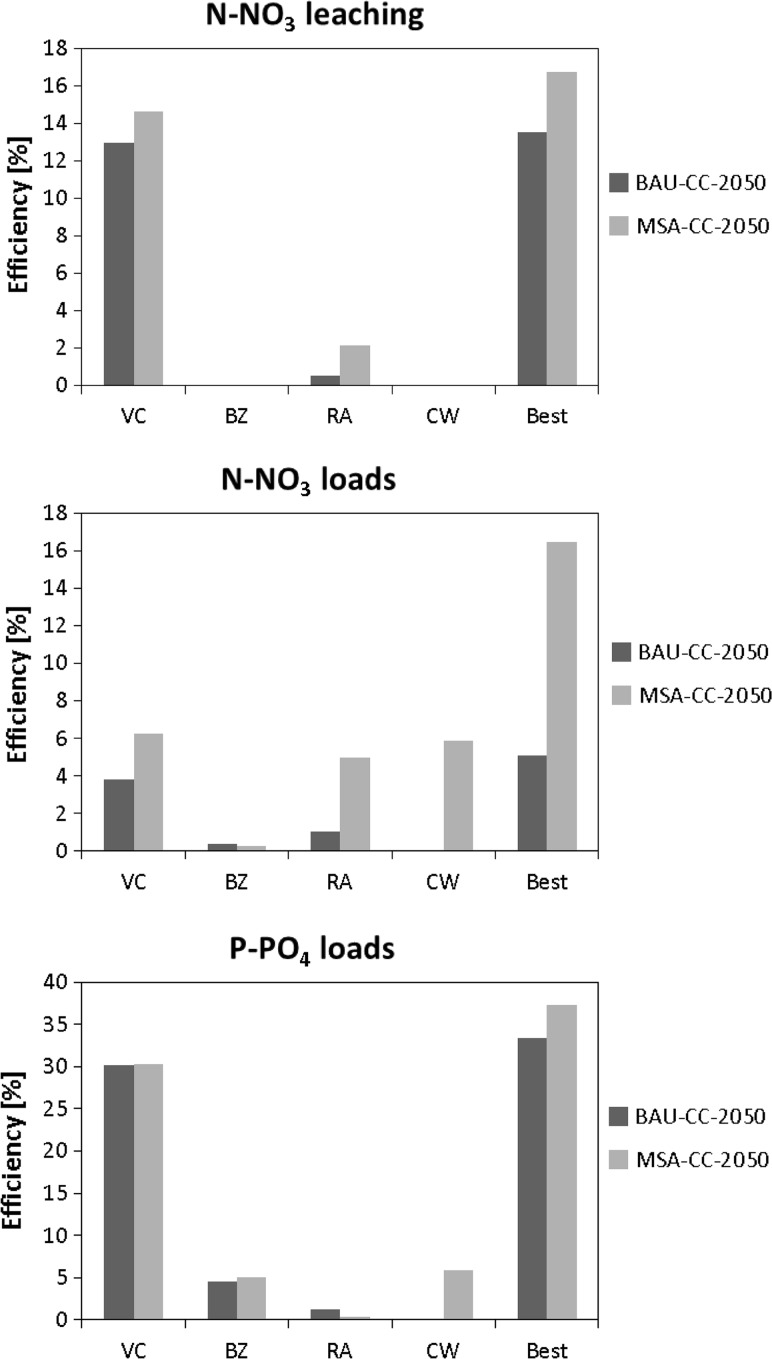
Simulated efficiencies of selected adaptation measures for different output variables under future scenarios (in percentage relative to a given future scenario, BAU-CC-2050 or MSA-CC-2050; *VC*—Vegetative cover in autumn and winter; *BZ*—Buffer zones along water areas and erosion-sensitive field areas; *RA*—Avoiding fertilization in risk areas; *CW*—Constructed wetlands for nutrient reduction/retention; *Best*—combination of VC, BZ, and RA for BAU-CC-2050 and of VC, BZ, RA, and CW for MSA-CC-2050)

Figure 7 shows calculated efficiencies in reduction of mean N-NO_3_ leaching to the shallow aquifer, of mean N-NO_3_ and P-PO_4_ loads at the watershed outlet for all studied single measures (VC, BZ, RA, and CW, the latter only for MSA-CC-2050), and for the most efficient combination of measures (VC + BZ + RA under BAU-CC-2050 and VC + BZ + RA + CW under MSA-CC-2050). Measures BZ and CW do not affect N-NO_3_ leaching to shallow aquifer at all, which can be explained by the way the model handles these measures. The only measure that has a measurable impact on N-NO_3_ leaching is VC. In the case of N-NO_3_ loads, however, its effect is dimmed since it does not affect N-NO_3_ in surface and subsurface runoff to such extent. Due to the low share of N-NO_3_ in surface runoff, measure BZ has a negligible effect on N-NO_3_ loads. Both measures VC and BZ are more efficient under MSA-CC-2050 than under BAU-CC-2050, whereas CW under MSA-CC-2050 has an efficiency similar to VC and BZ under this scenario. The results show that the best combination of measures is three times more efficient under MSA-CC-2050 than under BAU-CC-2050. The results are much different for P-PO_4_ loads. Measure VC has a very high efficiency under both BAU-CC-2050 and MSA-CC-2050, reaching 30 %, which is related to the anti-erosion function of plant cover in winter. Measures BZ and CW have a higher efficiency than for N-NO_3_ loads, while measure RA has negligible efficiency. Consequently, the efficiency of the best combination of measures is driven mainly by VC and is only slightly higher under MSA-CC-2050 than under BAU-CC-2050.

**Fig. 8:**
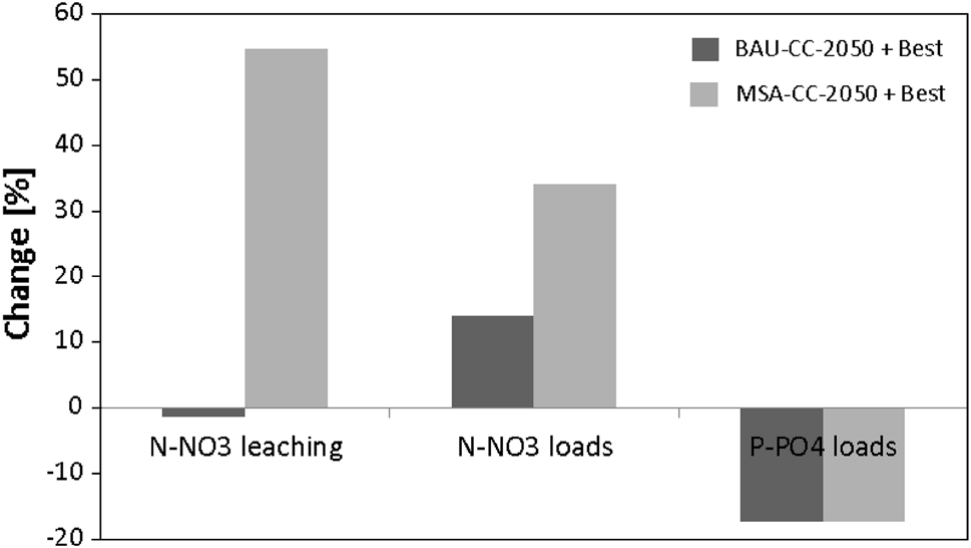
Percent change in selected variables between best combination of measures applied under future conditions and the baseline scenario

The last question to be answered was: What will be the combined effect (calculated using Eq. 3) of climate change, land use change, and of applying an adaptation measure, compared to the reference state. The answer to this question, illustrated in Fig. 8, depends on a variable and the scenario. Application of the best combination of measures would bring P-PO_4_ loads 17 % below the levels noted in the reference conditions both under BAU-CC-2050 and MSA-CC-2050, which emphasizes the importance of taking proper measures to counteract the expected negative effects. In the case of N-NO_3_ there is a noticeable difference between scenarios: under BAU-CC-2050 leaching to groundwater would be nearly the same as today, while under MSA-CC-2050 it would be 55 % higher. N-NO_3_ loads to the Puck Lagoon would in both cases be higher, by 14 and 34 %, respectively. This shows that the increase of N-NO_3_ loads that is triggered mainly by climate change in the case of BAU-CC-2050 and by agricultural intensification in the case of MSA-CC-2050 (cf. Fig. 5), would not be remediated by using even the most efficient combination of adaptation measures.

## Discussion

The results of this study concerning the projected impact of climate change on nutrient loads can be compared to those of similar studies carried out at the BSB level, despite scale and methodological differences. Meier et al. (2012) applied a statistical runoff model forced with a GCM-ensemble consisting of four transient simulations (one of the members was ECHAM5-RCA3-A1B used in this study) for the time period 2070–2099 in the whole BSB. They concluded that estimated nutrient loads in a future climate may increase due to increased runoff, in all projections and in all sub-basins. In contrast, Arheimer et al. (2012), who applied the same set of climate projections as Meier et al. (2012) but used a process-oriented Balt-HYPE model, found that N loads are expected to decrease under future climate conditions, pointing to the dilution effect of the higher water flow as the possible reason. Thus, our results correspond to those of Meier et al. (2012) and Neumann (2010); one should remember that local effects can be masked by large-scale aggregation. Furthermore, recent modeling studies in other geographical settings (20 large watersheds in the USA simulated using SWAT and HSPF) concluded that future streamflow and nutrient loads in many cases span a wide range and sometimes do not agree on the direction of change, resulting in uncertainty related to climate models and downscaling methodologies (U.S. EPA 2013).

Meier et al. (2012) also concluded that the climate effect in their study was larger than the effect of nutrient load reductions: even in the most optimistic scenario following the BSAP, reduction targets would not be achieved. In contrast, Arheimer et al. (2012) showed that for many climate projections, application of measures under future climate conditions could satisfy the BSAP targets. However, both of these studies ignored the fact that future nutrient loads might change considerably due to the effect of intensified agricultural production, which in Poland cannot be considered an unrealistic scenario. Our study demonstrates the importance of considering this driver in scenario modeling at the catchment scale. For example, under MSA-2050, the projected increase in N-NO_3_ loads is twice as large as under CC-2050. Consequently, even the most optimistic combination of measures applied under MSA-CC-2050 would not help to bring future N-NO_3_ loads below the levels observed today; even more important, future N-NO_3_ loads would be higher by 34 % than today’s levels, which show how difficult it would be to compensate for the negative effect of intensified production amplified by climate change.

It is noteworthy that agricultural intensification (as in MSA-2050) leads to more negative changes for N-NO_3_ than for P-PO_4_, whereas the adaptation measures are generally more efficient for the latter. This may be related to fundamental differences in the dominant transport pathways of these forms: in the Reda watershed the largest portion of nitrates leaches to the shallow aquifer, whereas leaching of P from the soil is neglected by SWAT and the dominant P transport pathway is sorption to sediment. Hence, our results concerning the effects of agricultural intensification as well as the applied measures will be compared to the results from other studies. It should be noted, though, that our measure efficiencies refer to the future and not present climate, and our results concern N-NO_3_ and P-PO_4_, while most other studies deal with TN and TP. It would be highly interesting to compare our measure efficiencies between the investigated N-NO_3_ and P-PO_4_ and non-investigated TN and TP, since the mechanisms influencing relative contribution of different N and P species to total loading tend to be complex (Heathwaite and Johnes 1996). Drawing conclusions on any other forms than N-NO_3_ and P-PO_4_ from our study would be highly speculative, since SWAT was not calibrated against these forms. It was calibrated for TSS, though, and in theory the calculated measure efficiencies for TSS could be a proxy for particulate N and P, but the Reda watershed is characterized by low magnitude and variability of TSS concentrations (mean 12.6 + SD 8.0 mg l^−1^; cf. Fig. S3 in the ESM) and low correlation between the organic forms of N and P and TSS (0.19 for organic N and 0.07 for organic P). Hence, the only reason why future measure efficiencies could be higher for TN and TP than for N-NO_3_ and P-PO_4_ is that under MSA-CC-2050 very high rates of organic fertilizers compared to the current ones would be applied.

Glavan et al. (2012), who applied SWAT to test the effect of land management scenarios on current nutrient loads in the UK watershed, emphasized the role of percolation and groundwater flow in N-NO_3_ transport: High reductions in nitrogen runoff were diminished at the river outlet level, as they were in our study. Regarding phosphorus, we found that a considerable increase in fertilizer rates under MSA-CC-2050 does not result in a dramatic increase in P-PO_4_ loads. Instead, surplus P is either removed in yield, which is much higher, or it builds up in the soil. This corresponds to the findings of Djodjic et al. (2004) who reported that despite high P fertilizing, P leaching has been found to be low in some investigated Swedish soils due to a high P sorption capacity in the subsoil. Thus, reduction of P losses after reducing P application can be delayed by many years compared to nitrogen (Bechmann et al. 2008). We also found that reducing fertilization to RA will have higher efficiency for N-NO_3_ than for P-PO_4_, as in the study of Lam et al. (2011) with SWAT in an agricultural watershed in northern Germany with high loads from diffuse sources.

Vegetative cover in autumn and winter (VC) is usually reported as a measure related to N rather than to P (Thorup-Kristensen and Nielsen 1998). We found that this measure was very efficient at the watershed scale for P-PO_4_ and quite efficient for N-NO_3_. Bechmann et al. (2008), who investigated long-term monitoring data from eight Norwegian agricultural watersheds, reported a very strong relationship between annual P concentrations and the area with a catch crop (*R* ^2^ = 0.93) and a much weaker relationship (*R* ^2^ = 0.49) between the annual N concentrations and the area with catch crops. They also found that erosion contributes more to the TP losses from cereal-dominated catchments than from the pasture-dominated catchments. In another SWAT study, researchers estimated that using catch crops as a measure to reduce N-NO_3_ losses was very efficient at the HRU scale and less efficient at the main outlet in a watershed situated in northwestern France (Laurent and Ruelland 2011).

Our study showed that the efficiency of BZ with respect to both N and P was somewhat lower than in previous studies with SWAT (Lam et al. 2011; Laurent and Ruelland 2011; Glavan et al. 2012); however, we used the model version in which a more physically based (though still simplified) representation of BZ had been implemented than in the mentioned papers, e.g., considering both sheet and concentrated overland flow conditions and evaluating BZ performance separately for particulate and non-particulate N and P (cf. White and Arnold 2009). The main uncertainty of modeling this measure with SWAT is related to appropriate parameterization and placement of BZ and whether the BZ in reality would trap only nutrients in surface runoff (as it is currently modeled in SWAT) or also in subsurface flow, e.g., by encouraging losses through denitrification (Heathwaite and Johnes 1996). Another important issue is that in SWAT HRUs are lumped within subwatersheds, while in reality in the Reda watershed grasslands often act as BZ trapping nutrient loads from arable land.

The efficiency of CW depends considerably on their proper dimensioning (Tattari et al. 2009) and on N and P settling velocities. As noted by Julich et al. (2009) the amount of removed nutrients in CW is rather roughly approximated by SWAT. In order to obtain meaningful results, we calibrated manually the settling velocities so that net reductions quantified in terms of kg of retained N or P per hectare of wetland were close to the upper limit of the range of values measured and modeled in 50 sites in Sweden (Weisner and Thiere 2010). The upper limit was chosen since it is expected that in Polish climate that is warmer than in Sweden, plant growth and nutrient uptake by plants in CWs would be higher. Simulated percent reductions in N-NO_3_ and P-PO_4_ under MSA-CC-2050 fall in the range reported by Tattari et al. (2009) for TN and TP in their study with SWAT in a small Finnish agricultural watershed.

## Conclusion

The results of this study demonstrate that both projected climate change and the level of intensity of agriculture are expected to have a strong effect on nutrient loads in a small-scale Polish watershed to the sea in the 2050s. We found that under projected warmer and wetter climatic conditions the discharge of water, N-NO_3,_ and P-PO_4_ to the sea would be ca. 20 % higher than at present. On the other hand, a shift in agriculture practices following the intensive Danish model would have a more pronounced effect than climate change on N-NO_3_ loads and a less pronounced effect than climate change on P-PO_4_ loads. In contrast, under the BAU scenario that is driven mainly by urban sprawl and a related increase in residential land cover, future levels of nutrient loads would remain nearly unchanged. If we consider that a climate change scenario is inevitable in the future, and that two land use change scenarios are the lower and upper limit of plausible change in agricultural practices in this coastal area, we could conclude that the range of increase in future N-NO_3_ and P-PO_4_ loads is from 20 to 60 %, and from 24 to 31 %, respectively. The simulated range of change in N-NO_3_ leaching to a shallow aquifer is even higher, from 14 to 86 %, which shows that different driving forces impact N-NO_3_ transport pathways in different ways. It should be stressed that up to date there were no studies that combined both climate and land use change for coastal watersheds in the BSB. However, changes in both driving forces that occur until 2050 are very uncertain, and the scenarios that we used are not comprehensive in considering all possible futures, so future work should focus on a better understanding, reducing and quantifying this uncertainty.

From the four adaptation measures that we considered, using vegetative cover in winter and spring (measure VC) would be the most efficient way to partly remediate the negative effects of climate change and a major shift in agricultural practices. Applying all the measures (VC, BZ, adapting fertilization, and CW) at one time would be even more efficient, especially in the case of P-PO_4_. However, in the case of N-NO_3_ we found that even the best combination of adaptation measures would not help to remediate the negative effects caused mainly by climate change (in the case of BAU-CC-2050) and by agricultural intensification (in the case of MSA-CC-2050). On the other hand, a major shift in Polish agriculture, following the Danish model, would bring significantly higher crop yields and a major deterioration in water quality. It also means that appropriate agricultural policy that encourages farmers to shift their attitude from pure profitability to profitability conditioned on environmental quality is of the highest importance for the Baltic Sea health.

In this study, we combined state-of-the-art watershed modeling with multidimensional scenario development to answer questions related to long-term projections of nutrient loads. We emphasized implementing realistic land use scenarios when modeling future, climate change-driven nutrient loads coming from watersheds. This study is an important contribution to the ongoing discussion on combating eutrophication in the Baltic Sea region. As such, it is relevant not only for this region but for all regions that face the choice between intensifying agriculture to improve their economy and conservation measures for protecting their environment.

We found that modeling of P-PO_4_ with SWAT is more challenging under Polish conditions than modeling N-NO_3_, presumably due to the higher mobility of nitrate, more complex P cycling in the soil, and because of the generally low magnitude and seasonal variability of measured P-PO_4_ concentrations. Therefore, future modeling studies in watersheds with dominant diffuse source pollution in this region should try to incorporate better local data on farming practices (including adaptation measures) as well as on soil P status. Regarding the scenario development process, in this study we use a large set of driving forces (e.g., climate, population, land use), but it was not exhaustive. Hence, future studies should attempt to add other aspects that seem relevant to the future of nutrient loads in this region, e.g., development of freshwater aquaculture, shale gas industry growth, or biofuel production.

## Electronic supplementary material

Below is the link to the electronic supplementary material.

Supplementary material 1 (PDF 1436 kb)

## Footnotes

1
http://www.balticcompass.org/_blog/Project_Reports/post/Prioritized_measures_by_Work_Package_3_/ (last accessed 3/28/2013).

2
http://baltyk.imgw.gdynia.pl/warunki/odplyw_zanieczyszczen.html (last accessed 3/28/2013).
