# Supplementary material for: Climate Change and Agricultural Development: Adapting Polish Agriculture to Reduce Future Nutrient Loads in a Coastal Watershed
Source: Ambio. 2013 Oct 24;43(5):644–60. doi: 10.1007/s13280-013-0461-z (PMC4132465; doi:10.1007/s13280-013-0461-z)
Supplement: Supplementary file 1 — Supplementary material 1 (PDF 1436 kb) [file 13280_2013_461_MOESM1_ESM.pdf]

Electronic supplementary material

Climate Change and Agricultural Development: Adapting Polish Agriculture to Reduce Future Nutrient Loads in a Coastal Watershed

Mikołaj Piniewski, Ignacy Kardel, Marek Giełczewski, Paweł Marcinkowski, Tomasz Okruszko

This supplementary material contains the following information:

A. SWAT model
B. Model calibration approach
C. Calculation of residential land cover increase in SWAT
D. Calibration and validation results

A. SWAT model

SWAT is a physically based, semi-distributed, continuous-time model that operates on a daily time step and simulates the movement of water, sediment, and nutrients on a watershed scale. The smallest unit of discretization is a unique combination of land use, soil, and slope overlay, referred to as a “hydrological response unit” (HRU). Runoff is predicted separately for each HRU and then aggregated to the sub-basin level and routed through the stream network to the main outlet in order to obtain the total runoff for the river basin.

No matter what type of problem one studies with SWAT, water balance is the driving force behind everything that happens in the watershed. For this study, we selected the modified USDA Soil Conservation Service (SCS) curve number method for calculating surface runoff and the Penman-Monteith method for estimating potential evapotranspiration. Channel routing was modeled using a variable storage coefficient approach. SWAT uses the degree-day method for snowmelt estimation.

SWAT uses a plant growth model to simulate all types of land covers that is based on EPIC (Williams, 1990). The plant growth model is used to assess removal of water and nutrients from the root zone, transpiration, and biomass/yield production. Erosion and sediment yield are calculated for each HRU using the Modified Universal Soil Loss Equation (MUSLE) (Williams and Berndt 1977), which uses the amount of runoff as an indicator of erosive energy. Sediment transport in the channel network is a function of two processes, deposition and degradation. The occurrence of these processes is determined by the stream power, the exposure of a channel’s sides and bottom to the erosive force of the stream and the composition of channel bank and bed sediment.

SWAT tracks the movement and transformation of several forms of nitrogen and phosphorus in the watershed. In the soil, the model simulates principal processes included in the nutrients cycle that control the transformation of nutrients from one form to another. In the nitrogen cycle, the main processes are: denitrification, nitrification, mineralization, plant uptake, decay, fertilization, volatilization, and in the phosphorus cycle they are: mineralization, fertilization, decay, and plant uptake. Nutrients may be introduced to the main channel and transported downstream through surface runoff and lateral subsurface flow. The in-stream water quality component allows the researcher to control nutrient transformations in the stream. The in-stream kinetics used in SWAT for nutrient routing are adapted from QUAL2E (Brown and Barnwell 1987). The model tracks two pools of nutrients: those dissolved in the stream and those adsorbed to the sediment. Dissolved nutrients are transported with the water while those adsorbed to sediments are allowed to be deposited with the sediment on the bed of the channel.

B. Model calibration approach

SWAT is a river-basin scale model, in which hydrological cycle drives the water, sediment and nutrient movement. Due to a large number of parameters, SWAT requires conducting calibration which includes fitting simulations to observations, usually by using automatic routines of various types. Calibration should be preceded by the sensitivity analysis which measures the response of model outputs and objective function to altering input parameter values. Model validation is usually conducted using calibrated parameter values for a different time period than the one used in calibration. In this study, the entire process consisting of sensitivity analysis, calibration and validation was conducted in four iterations related to different variables simulated by the model:

1. Discharge
    a. Sensitivity analysis of parameters potentially affecting discharge
    b. Selection of calibration parameters
    c. Calibration (time period 1998-2002)
    d. Validation (time period 2003-2006)
    e. Writing best fit parameter values into the SWAT project
2. Total suspended sediment (TSS)
    a. Sensitivity analysis of parameters potentially affecting TSS load (excluding the parameters selected in point 1b)
    b. Selection of calibration parameters
    c. Calibration (time period 1998-2002)
    d. Validation (time period 2003-2006)
    e. Writing best fit parameter values into the SWAT project
3. Nitrate nitrogen (N-NO_3)
    a. Sensitivity analysis of parameters potentially affecting N-NO_3 load (excluding the parameters selected in point 1b and 2b)
    b. Selection of calibration parameters
    c. Calibration (time period 1998-2002)
    d. Validation (time period 2003-2006)
    e. Writing best fit parameters into the SWAT project
4. Mineral phosphorus (P-PO_4)
    a. Sensitivity analysis of parameters potentially affecting P-PO_4 load (excluding the parameters selected in point 1b, 2b and 3b)
    b. Selection of calibration parameters
    c. Calibration (time period 1998-2002)
    d. Validation (time period 2003-2006)
    e. Writing best fit parameters into the SWAT project

Simulation of nutrient loads is significantly conditioned by the hydrological cycle and thus the accuracy of calculations depends on the properly conducted calibration of discharge. The aim of this stage of calibration is to reflect spatial and temporal variability of water balance in the analyzed watershed and to satisfactorily reproduce variability of daily streamflow.

Daily discharge time series from three gauging stations (the River Bolszewka at Bolszewo and the River Reda at Zamostne and Wejherowo, cf. Fig. 1 of the manuscript) were acquired from the Institute of Meteorology and Water Management – National Research Institute in order to perform step 1 from the aforementioned list. The following three steps concerned calibration of sediment, nitrate and mineral phosphorus loads, based on the dataset acquired from the General Inspectorate of Environmental Protection in Gdańsk (GIOŚ), containing bimonthly measurements of sediment and

nutrient concentrations of the Reda at Wejherowo between 1998 and 2006. Average daily loadings (kg/day) of selected water quality parameters were calculated based on daily discharge data (m^3/day) at Wejherowo gauging station.

The SWAT-CUP program (Abbaspour 2008) was used for sensitivity analysis, calibration and validation. The main function of this tool is to provide a link between the input/output of a calibration program and the SWAT model through iterative altering of parameters values (selected beforehand during sensitivity analysis) using SUFI-2 (Sequential Uncertainty Fitting Version 2) algorithm that combines optimization with uncertainty analysis. The calibration process is considered as successfully completed when the satisfactory values of defined objective function (e.g. Nash-Sutcliffe efficiency – NSE; Moriasi et al. 2007), *p*-factor and *r*-factor (uncertainty parameters) are obtained. The *p*-factor represents the percentage of measured data bracketed by a 95% prediction uncertainty (95PPU), and the *r*-factor quantifies the average thickness of the 95PPU uncertainty band divided by the standard deviation of the measured data. In this study NSE was used as an objective function for each of the model outputs, however, we kept track of other goodness-fit values (coefficient of determination R^2, percent bias PBIAS) and uncertainty indicators (*p*-factor, *r*-factor).

Each calibration/validation model run was performed with a warm-up period of three years, i.e. the simulation start was set to 1 January 1995. This allowed stabilizing the initial soil moisture content and soil nitrogen and phosphorus pools.

C. Calculation of residential land cover increase in SWAT

The urban SWAT land cover type that represents low density residential land is called a URLD (cf. Fig. 1C). Future increases in the area covered by this class would be at the expense of fallow land (FALL) and the lowest quality agricultural land (called GO7, where rye is typically grown). The projected future increment in the area covered by the URLD class between 2050 and reference conditions ΔA [ha] was calculated as:

$$\Delta A = \frac{N \cdot \alpha}{M} \cdot F, \quad (1)$$

where N denotes the population living in the area covered by URLD in the reference conditions, α denotes projected population growth between 2010 and 2050, M denotes the average number of people living in one household in the URLD class, and F denotes the average residential farm size covered by 1 household [ha]. The values of N, α, M and F were estimated to be 97 000, 37%, 4, and 0.1 ha, respectively, based on available statistical data, commune authorities' data, and stakeholder opinions. Hence, the calculated value of ΔA yielded 909 ha, which is 30% of the current area of a URLD. In the final step, the estimated value of ΔA was disaggregated into SWAT sub-basins assuming that growth in residential areas will be proportional to a percentage of marginal (classes FALL and GO7) land in the current state and will occur only in sub-basins in which the URLD class exists in the current state. Technically, in SWAT this type of land cover change was represented by a manipulation of the parameter HRU_FR (fraction of HRU within sub-basin).

D. Calibration and validation results

Figures S1 and S2 illustrate simulated versus observed flows, TSS load, N-NO_3 load and P-PO_4 load, for the calibration and validation periods, respectively. The graphs illustrating variability of discharge are presented with daily time step, whereas other graphs with bimonthly time step. The assessment

criteria of hydrological model performance are varied in literature. For the SWAT model the most commonly used criteria were developed by Moriasi et al. (2007). According to these criteria, simulation of daily discharge by SWAT can be assessed as good. Both NSE and R^2 exceed 0.7 in both calibration and validation period and percent bias does not exceed 10% (cf. Tab. 1 of the manuscript). The underestimation of runoff occurs usually in the first quarter of the year (January - March) which is the high flow period, with frequent snowmelt and rain events.

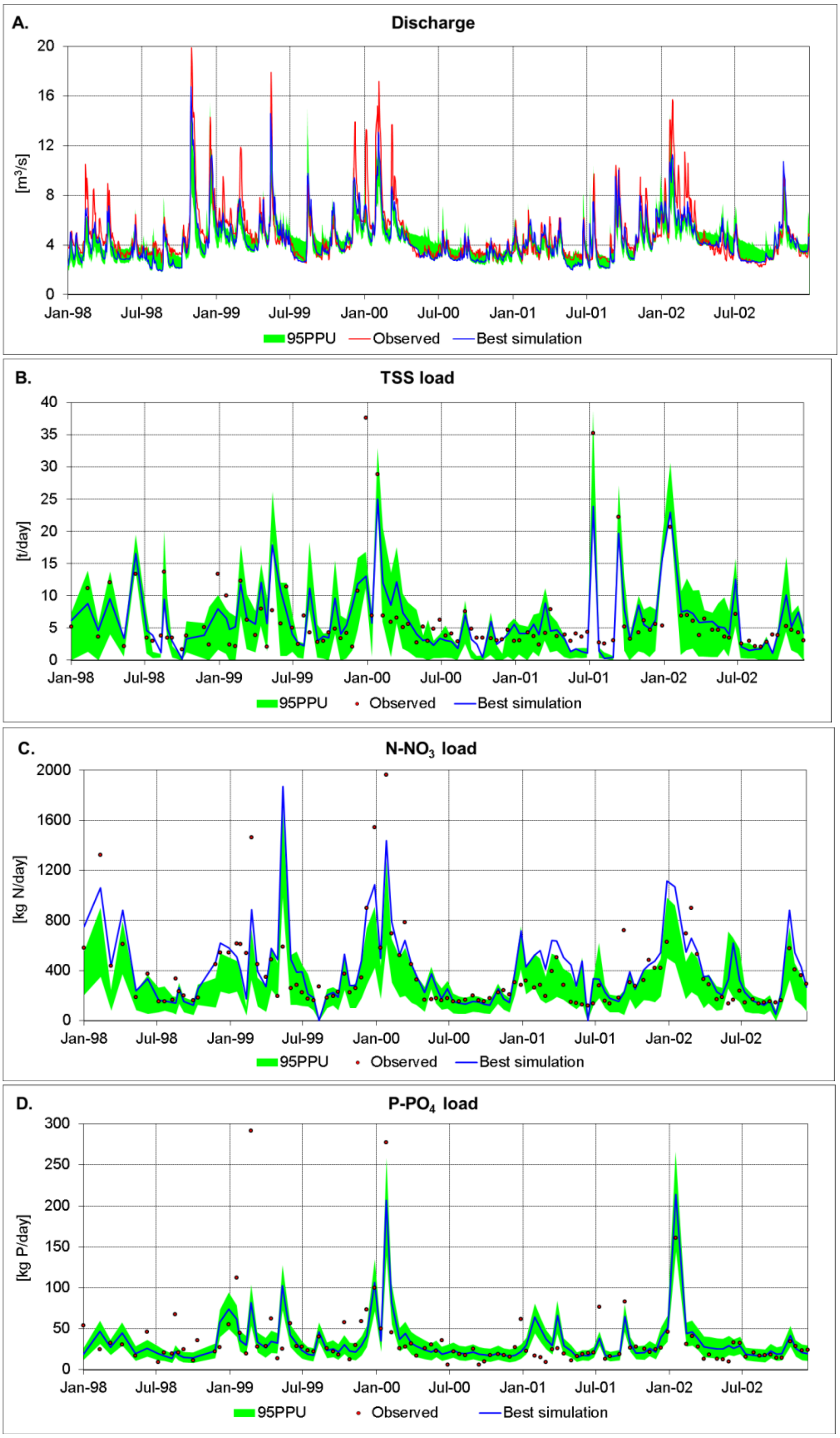

Fig. S1 Calibration plots for discharge (A), TSS load (B), N-NO_3 load (C) and P-PO_4 load (D) – time period 1998-2002.

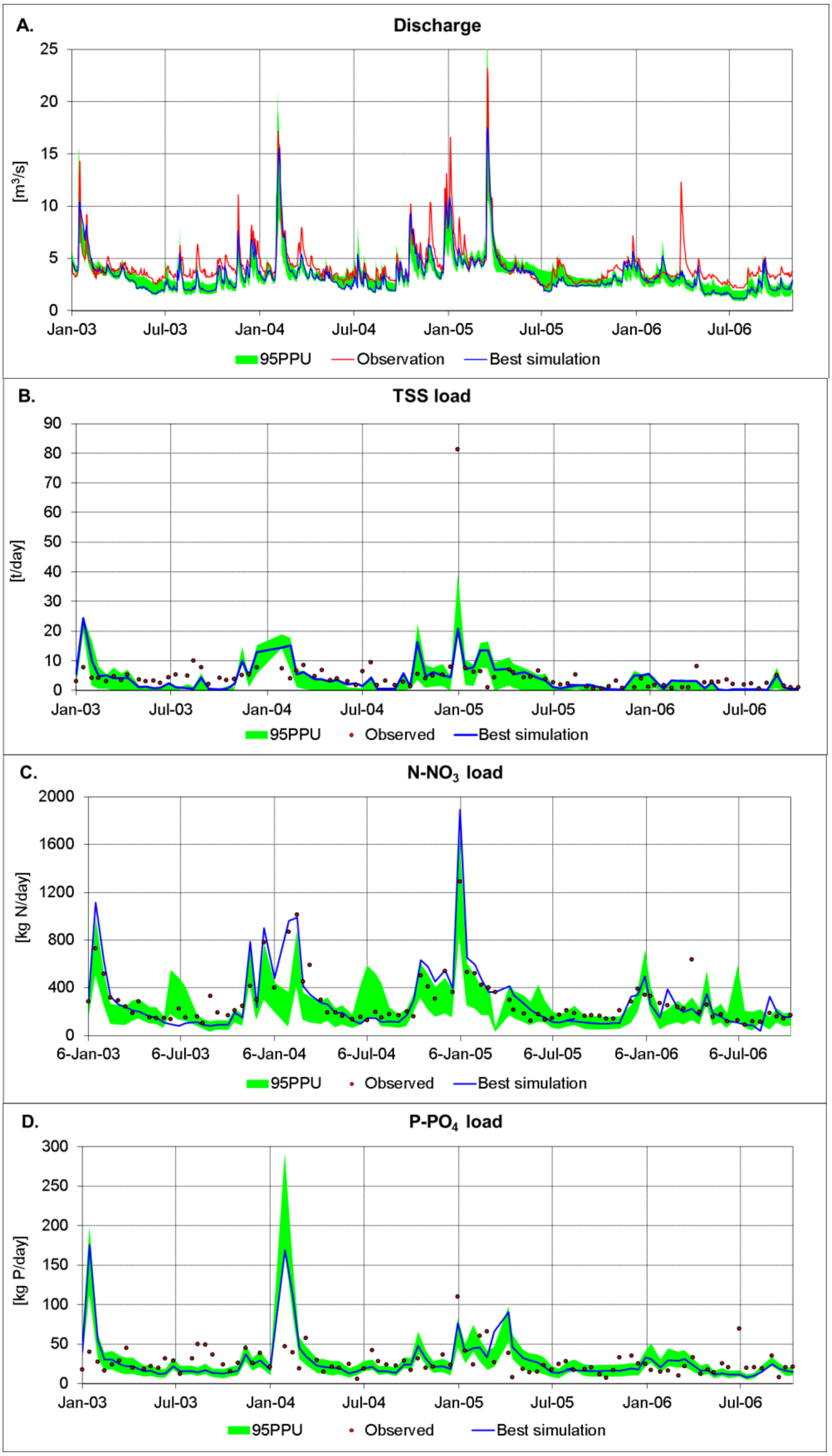

Fig. S2 Validation plots for discharge (A), TSS load (B), $N\text{-}NO_3$ load (C) and $P\text{-}PO_4$ load (D) – time period 2003-2006.

Figures S1 and S2 present discharge simulation results only for the Reda at Wejherowo, whereas two other flow gages situated upstream of Wejherowo (Fig. 1A of the manuscript) were used as well in calibration and validation. The goodness-of-fit measures were only a little worse for those stations than for Wejherowo; NSE and R^2 were equal to 0.58 and 0.65 for Zamostne and 0.60 and 0.60 for Bolszewo.

The Reda watershed is characterized by the highest mean specific runoff (*q,* mean discharge per unit area) in the Polish Plain, exceeding 10 l/s/km^2. (Stachý and Biernat 1987; Bogdanowicz et al. 2007). Furthermore, *q* exhibits a clear gradient from the seaside (5-6 l/s/km^2) towards the upland (12-15 l/s/km^2). A similar scale of spatial variability was obtained as a result of modeling in SWAT.

Another feature of the Reda watershed is very high (compared to other watersheds in the Polish Plain) contribution of groundwater in total runoff. According to the map of groundwater contribution to streamflow, two classes are present in the Reda watershed: 60-75% and above 75% (Orsztynowicz, 1988). A SWAT-based estimate equals 69%, which shows a good match with the map of Orsztynowicz (1988). The model performed reasonably well in simulation of low flow magnitude. Low flows are exceptionally high in the Reda watershed (simulated and observed mean annual minimum specific runoff equal to 5.4 and 6.2 l/s/km^2, respectively) and discharge variability is also exceptionally low. Modeled and observed coefficient of variation of daily flows equaled to 0.47 and 0.48, respectively.

Simulation of N-NO_3 load is good, which is reflected by relatively high values of performance measures (NSE for calibration and validation period reached 0.62 and 0.64, respectively) and visual inspection of plots in Figures S1C and S2C. It is noteworthy that SWAT correctly simulates seasonal variability of N-NO_3 load: The highest values are observed during winter and the lowest during summer. Observed N-NO_3 concentrations are strongly correlated with discharge (R^2 equal to 0.45 for the time period 1998-2006). The mass balance of transported nitrate load is well conserved – PBIAS for calibration and validation periods is equal to -4% and 3%, respectively.

Simulation results for sediment load are worse than those for discharge and N-NO3 loads, especially during validation period (Fig. S2B). It is noteworthy that the temporal variability of sediment load transported through the Reda river at Wejherowo gaging station is relatively low, which is determined by low variability of discharge and generally low TSS concentration (the values exceeding 50 mg/l occurred only twice per 206 observations carried out in years 1998-2006).

The results of P-PO_4 calibration are good for calibration period and poor for validation period. NSE for calibration period equals 0.53, whereas for validation period -1.78. This poor result is partly influenced by unsatisfactory TSS load simulation during validation period, as significant amount of P-PO_4 is transported with sediment. Another possible reason for such a large difference between calibration and validation might be the lack of homogeneity of observed data between two periods. Both the loads and concentrations of P-PO_4 demonstrate smaller seasonal variability compared to corresponding loads and concentrations of N-NO_3. For example, correlation between P-PO_4 concentration and discharge was observed only for the calibration period (R^2=0.15, compared to 0 for validation period). Figure S3 illustrates monthly variability of the observed mean TSS, N-NO_3 and P-PO_4 concentrations in calibration and validation periods. In the calibration period maximum values of P-PO_4 concentration occurred in winter, whereas in the validation period in summer. Also for TSS monthly variability differed to a large extent between two periods. In contrast, N-NO_3 concentrations were quite similar in both periods. Hence, it is argued that these lack of homogeneity explains observed differences in goodness-of-fit measures in validation period, that were good for N-NO_3 and

poor for sediment and P-PO_4. The model was not able to simulate accurately maximum P-PO_4 loads observed in summer months, whereas in winter of 2003 and 2004 maximum loads were simulated by the model despite not being present in measurements. It is noteworthy, however, that the mass balance of P-PO_4 was simulated accurately (percent bias for calibration and validation periods yielded -2% and 5%, respectively).

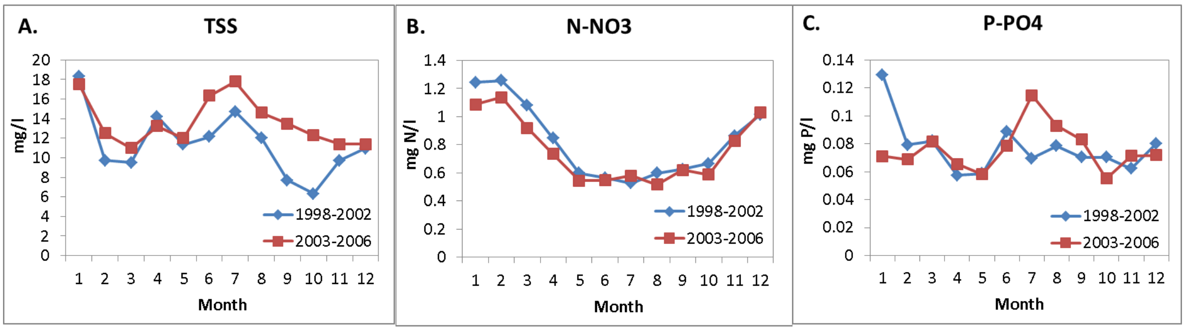

Fig. S3 Observed mean monthly concentrations of TSS (A), N-NO_3 (B) and P-PO_4 for calibration (1998-2002) and validation (2003-2006) periods.

The plots shown in Figures S1 and S2 illustrate the best fit simulation results against the observations as well as the 95PPU uncertainty band corresponding to the final parameter ranges obtained in SUFI-2 (Tab. S1-S4). In addition the uncertainty measures were presented separately in Figure S4**Error! Reference source not found.**, which shows that *p*-factor reached similar values for all variables (75-77%) in calibration period. The variability of *r*-factor was significantly higher: high for sediment (1.06) compared to the rest of variables (0.61-0.72). During validation period the uncertainty measures were slightly worse than in calibration period for discharge and significantly worse for P-PO_4.

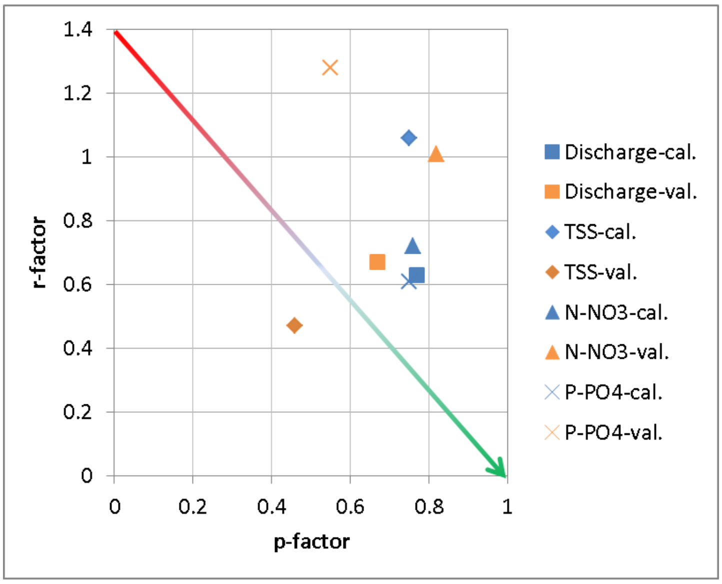

Fig. S4 Plot of two uncertainty measures (*p*-factor against *r*-factor) for different model outputs in calibration and validation periods. The arrow direction (red-green gradient) indicates decreasing uncertainty reflected by increasing *p*-factor and decreasing *r*-factor.

Tab. S1 Fitted parameter values and optimal parameter ranges calculated using SUFI-2 during calibration of discharge.

Parameter name[1]	Definition	Fitted value	Min value	Max value
r__CN2.mgt	Initial SCS runoff curve number for moisture condition II (-)	-0.038	-0.10	0.01
v__GW_DELAY.gw	Groundwater delay time (days)	397	250	500
r__GWQMN.gw	Threshold depth of water in the shallow aquifer required for return flow to occur (mm H2O)	-0.14	-0.8	0.5
r__REVAPMN.gw	Threshold depth of water in the shallow aquifer for revap or percolation to the deep aquifer to occur (mm H2O)	-0.32	-0.5	1
r__SOL_Z.sol	Depth from soil surface to bottom of layer (mm)	0.06	-0.35	0.2
r__SOL_BD().sol	Moist bulk density (g/cm^3)	0.36	-0.1	0.4
r__GW_REVAP.gw	Groundwater "revap" coefficient (-)	0.46	-0.6	0.6
v__ESCO.hru	Soil evaporation compensation factor (-)	0.85	0.82	1
v__TIMP.bsn	Snow pack temperature lag factor (-)	0.36	0.2	0.8
r__SOL_K().sol	Saturated hydraulic conductivity (mm/hr)	-0.38	-0.8	0
r__HRU_SLP.hru	Average slope steepness (m/m)	-0.02	-0.45	0.1
v__EPCO.hru	Plant uptake compensation factor (-)	0.02	0	0.5
v__SURLAG.bsn	Surface runoff lag coefficient (-)	0.1	0.05	0.45
r__RCHRG_DP.gw	Deep aquifer percolation fraction (-)	-0.05	-0.8	0.8
r__SLSUBBSN.hru	Average slope length (m)	0.02	-0.05	0.4

[1] 'r__' – indicates relative change; 'v__' – indicates replacement by a new value; suffixes '.gw', '.swq, etc. – SWAT file extensions.

Tab. S2 Fitted parameter values and optimal parameter ranges calculated using SUFI-2 during calibration of TSS load.

Parameter name[1]	Definition	Fitted value	Min value	Max value
r__USLE_K.sol	USLE equation soil erodibility (K) factor (-)	0.52	0.3	0.7
v__CH_COV2.rte	The channel cover factor (-)	1.92	1.9	3
r__CH_N2.rte	Manning's "n" value for the main channel (-)	0.62	0.42	0.62
v__RES_D50.res	Median particle diameter of sediment in reservoir (µm)	7.76	2	8

[1] 'r__' – indicates relative change; 'v__' – indicates replacement by a new value; suffixes '.sol', '.rte', etc. – SWAT file extensions.

Tab. S3 Fitted parameter values and optimal parameter ranges calculated using SUFI-2 during calibration of N-NO_3 load.

Parameter name[1]	Definition	Fitted value	Min value	Max value
v__CDN.bsn	Denitrification exponential rate coefficient (-)	0.004	0	0.3
v__RCN.bsn	Concentration of nitrogen in rainfall (mg/l)	1.73	1.5	1.75
v__CMN.bsn	Rate factor for humus mineralization of active organic nutrients (-)	0.0021	0.002	0.0024
v__SDNCO.bsn	Denitrification threshold water content (-)	0.941	0.94	0.96
v__NPERCO.bsn	Nitrate percolation coefficient (-)	0.71	0.7	0.87
v__RSDCO.bsn	Residue decomposition coefficient (-)	0.058	0.05	0.07
r__SOL_NO3.chm	Initial NO3 concentration in the soil layer (mg N/kg soil, dry weight)	-0.01	-0.1	0.05
v__SOL_ORGN.chm	Initial organic N concentration in the soil layer (mg N/kg soil, dry weight)	578.9	570	640
v__BC3.swq	Rate constant for hydrolysis of organic N to NH4 in the reach at 20º C (day^{-1})	0.34	0.32	0.35
v__AI1.wwq	Fraction of algal biomass that is nitrogen (-)	0.0754	0.075	0.081
v__HLIFE_NGW.gw	Half-life of nitrate in the shallow aquifer (days)	1.73	1	4
v__BIOMIX.mgt	Biological mixing efficiency (-)	0.34	0.3	0.44

[1] 'r__' – indicates relative change; 'v__' – indicates replacement by a new value; suffixes '.sol', '.rte', etc. – SWAT file extensions.

Tab. S4 Fitted parameter values and optimal parameter ranges calculated using SUFI-2 during calibration of P-PO_4 load.

Parameter name[1]	Definition	Fitted value	Min value	Max value
v__RSDIN.hru	Initial residue cover (kg/ha)	5676	3894	6039
v__PPERCO.bsn	Phosphorus percolation coefficient (m^3/Mg)	12.52	11.78	13.49
v__PSP.bsn	Phosphorus availability index (-)	0.189	0.13	0.26
v__RSDCO.bsn	Residue decomposition coefficient (-)	0.074	0.047	0.088
v__BC4.swq	Rate constant for mineralization of organic P to dissolved P in the reach at 20º C (day^{-1})	0.345	0.26	0.41
r__SOL_SOLP().chm	Initial soluble P concentration in soil layer (mg P/kg soil, dry weight)	-0.137	-0.15	0.05
v__SOL_ORGP().chm	Initial humic organic phosphorus in soil layer (mg P/kg soil, dry weight)	343	275	358
v__ERORGP.hru	Phosphorus enrichment ratio for loading with sediment (-)	1.70	1.29	2.22
v__GWSOLP.gw	Concentration of soluble phosphorus in groundwater contribution to streamflow from subbasin (mg P/l)	0.070	0.03	0.116
v__AI2.wwq	Fraction of algal biomass that is phosphorus	0.013	0.011	0.0132
v__CH_OPCO.rte	Organic phosphorus concentration in the channel (mg/l)	38.8	23	41
v__MUMAX.wwq	Maximum specific algal growth rate at 20º C (day^{-1})	1.73	1.61	2.07
v__RHOQ.wwq	Algal respiration rate at 20º C (day^{-1})	0.350	0.29	0.382

[1] 'r__' – indicates relative change; 'v__' – indicates replacement by a new value; suffixes '.sol', '.rte', etc. – SWAT file extensions.

References

Abbaspour, K. 2008. SWAT-CUP2: SWAT Calibration and Uncertainty Programs - a user manual. Department of Systems Analysis, Integrated Assessment and Modelling (SIAM), Eawag, Swiss Federal Institute of Aquatic Science and Technology, Duebendorf, Switzerland, 95pp.

Bogdanowicz R., R. Cieśliński, J. Drwal, and A. Cysewski. 2007. Estimation of the local riverine pollution load to the lagoon on the southern Baltic coast (the Puck Lagoon). Sowriemiennyj naucznyj wiestnik 5: 28-36.

Brown, L.C., and T.O. Barnwell. 1987. The Enhanced Stream Water Quality Models QUAL2E and QUAL2E-UNCAS: Documentation and User Manual. Cooperative Agreement No. 811883,

Environmental Research Laboratory, Office of Research and Development, U.S. Environmental Protection Agency, Agency, Georgia.

Moriasi, D. N., J. G. Arnold, M.W. van Liew, R.L. Bingner, R.D. Harmel, and T.L. Veith. 2007. Model evaluation guidelines for systematic quantification of accuracy in watershed simulations. Transactions of the Asabe 50(3): 885–900.

Orsztynowicz, J. 1988. Studium naukowo-badawcze do Atlasu hydrogeologicznego Polski. Średnie roczne i wieloletnie odpływy podziemne na obszarze Polski w okresie 1951–1980. IMiGW, Zakład Dynamiki Wód Podziemnych, Warszawa (in Polish).

Stachý, J. and B. Biernat. 1987. Odpływ rzeczny. Średni odpływ jednostkowy [In:] Stachý, J. (ed.). Atlas Hydrologiczny Polski, IMGW, Wyd. Geol., Warszawa, s. 56 (in Polish).

Williams, J.R. 1990. The Erosion-Productivity Impact Calculator (EPIC) Model: A Case History. Philosophical Transactions: Biological Sciences. 329(1255): 421-428.

Williams, J.R., and H.D. Berndt. 1977. Sediment yield prediction based on watershed hydrology. Trans. Am. Soc. Agric. Eng. 20: 1100-1104.
